# Recommendations for Determining the Validity of Consumer Wearables and Smartphones for the Estimation of Energy Expenditure: Expert Statement and Checklist of the INTERLIVE Network

**DOI:** 10.1007/s40279-022-01665-4

**Published:** 2022-03-09

**Authors:** Rob Argent, Megan Hetherington-Rauth, Julie Stang, Jakob Tarp, Francisco B. Ortega, Pablo Molina-Garcia, Moritz Schumann, Wilhelm Bloch, Sulin Cheng, Anders Grøntved, Jan Christian Brønd, Ulf Ekelund, Luis B. Sardinha, Brian Caulfield

**Affiliations:** 1 Insight Centre for Data Analytics, University College Dublin, Dublin, Ireland; 2 School of Public Health, Physiotherapy and Sport Science, University College Dublin, Dublin, Ireland; 3 School of Pharmacy and Biomolecular Sciences, Royal College of Surgeons in Ireland, Dublin, Ireland; 4 Exercise and Health Laboratory, CIPER, Faculdade de Motricidade Humana, Universidade de Lisboa, Lisbon, Portugal; 5 Department of Sport Medicine, Norwegian School of Sport Sciences, Oslo, Norway; 6 PROFITH (PROmoting FITness and Health Through Physical Activity) Research Group, Department of Physical Education and Sports, Faculty of Sport Sciences, University of Granada, Granada, Spain; 7 Department of Bioscience and Nutrition, Karolinska Institutet, Solna, Sweden; 8 Institute of Cardiovascular Research and Sports Medicine, Department of Molecular and Cellular Sports Medicine, German Sport University, Cologne, Germany; 9 Exercise Translational Medicine Centre, the Key Laboratory of Systems Biomedicine, Ministry of Education, and Exercise, Health and Technology Centre, Department of Physical Education, Shanghai Jiao Tong University, Shanghai, China; 10 Faculty of Sport and Health Sciences, University of Jyväskylä, Jyväskylä, Finland; 11 Department of Sports Science and Clinical Biomechanics, Research Unit for Exercise Epidemiology, Centre of Research in Childhood Health, University of Southern Denmark, Odense M, Denmark

## Abstract

**Background:**

Consumer wearables and smartphone devices commonly offer an estimate of energy expenditure (EE) to assist in the objective monitoring of physical activity to the general population. Alongside consumers, healthcare professionals and researchers are seeking to utilise these devices for the monitoring of training and improving human health. However, the methods of validation and reporting of EE estimation in these devices lacks rigour, negatively impacting on the ability to make comparisons between devices and provide transparent accuracy.

**Objectives:**

The Towards Intelligent Health and Well-Being Network of Physical Activity Assessment (INTERLIVE) is a joint European initiative of six universities and one industrial partner. The network was founded in 2019 and strives towards developing best-practice recommendations for evaluating the validity of consumer wearables and smartphones. This expert statement presents a best-practice validation protocol for consumer wearables and smartphones in the estimation of EE.

**Methods:**

The recommendations were developed through (1) a systematic literature review; (2) an unstructured review of the wider literature discussing the potential factors that may introduce bias during validation studies; and (3) evidence-informed expert opinions from members of the INTERLIVE network.

**Results:**

The systematic literature review process identified 1645 potential articles, of which 62 were deemed eligible for the final dataset. Based on these studies and the wider literature search, a validation framework is proposed encompassing six key domains for validation: the target population, criterion measure, index measure, testing conditions, data processing and the statistical analysis.

**Conclusions:**

The INTERLIVE network recommends that the proposed protocol, and checklists provided, are used to standardise the testing and reporting of the validation of any consumer wearable or smartphone device to estimate EE. This in turn will maximise the potential utility of these technologies for clinicians, researchers, consumers, and manufacturers/developers, while ensuring transparency, comparability, and replicability in validation.

**Trial Registration:**

PROSPERO ID: CRD42021223508.

**Supplementary Information:**

The online version contains supplementary material available at 10.1007/s40279-022-01665-4.

## Key Points

**Table:** 

This systematic literature review of validation studies of consumer wearables and smartphone applications has highlighted a heterogeneity between validation methodologies in key domains, particularly in the target populations, data processing and statistical approaches, giving rise to validation bias.
The lack of free-living validation leads to limited abilities of users to understand the accuracy of wearable devices and smartphones in estimating energy expenditure (EE) in the intended use case.
In this article, the INTERLIVE network provides best-practice recommendations to be used in future protocols to move towards a more accurate, transparent and comparable validation of EE estimation derived from consumer wearables and smartphone applications.

## Introduction

Worldwide, the number of consumer wearable devices sold in 2020 is estimated to have reached almost 400 million units [1]. The growing popularity of these devices, combined with the number of smartphone users today surpassing 3 billion [2], means that more people than ever are able to self-monitor their physical health and activity. Additionally, consumer wearables are being increasingly utilised in healthcare and research settings [3]. These devices can assess a variety of metrics associated with physical activity including step count, heart rate, maximal oxygen consumption (*V*O_2max_), and energy expenditure (EE).

Traditionally, the measurement of EE has been conducted via indirect calorimetry (where gaseous exchange is measured and converted to EE using standard formulae such as the Weir Equation [4]), direct calorimetry (the measurement of heat production by the body), or doubly labelled water (DLW; where urine samples are analysed using isotope ratio mass spectrometry) [5, 6]. While DLW is considered the gold standard for measuring free-living EE [6, 7], it only provides a measure of total EE (TEE), and the active EE (AEE) is determined by subtracting an estimate of the resting EE. Additionally, DLW is an expensive and complex approach, requiring specialist skills and resources. Therefore, indirect calorimetry has been the predominant criterion measure for AEE in recent years [6]. However, these indirect calorimetry methodologies are limited to more controlled protocols requiring the use of metabolic carts, which are expensive and require technical skills to administer.

The widespread availability, relatively low cost, and wearability of consumer activity monitors means that data that could only be captured in specialist settings are now available to the general population, and these devices are increasingly being used to provide measures of EE. Most users seek this information for either AEE in monitoring calorie consumption during a specific exercise activity, or to support them with their TEE across a day, possibly in the management of diet or weight loss. A variety of inputs, including user demographics such as age, sex, weight and height, and data from on-board sensors, including accelerometers and/or photoplethysmography heart rate sensors, are used to make estimates of EE [8–10]. However, the validity and reliability of consumer wearables to estimate group- and individual-level EE is subject to debate, with numerous validation studies being conducted, offering varying methodologies and results [11, 12]. The inconsistency in validation protocols used in the literature, along with a lack of transparency in reporting standards means that it is difficult for consumers and clinicians to navigate the market and understand or compare the accuracy of such devices [13]. Whether a consumer monitor can be considered accurate for estimating EE is determined by a combination of the target population, the intended output (daily TEE, daily AEE, training session EE, training session AEE, etc.) and the transferability of the validation protocol to the intended use. For example, a consumer monitor with reported high accuracy during an incremental walking or running protocol may not be the right choice for an individual who expends energy above rest, primarily during less formal activities. As consumer monitors will be used for a range of purposes, a comprehensive evaluation during discrete activities and free-living settings is needed to maximise applicability.

There have been calls for standardisation of the evaluation of consumer wearable devices, with requests for marketing claims from manufacturers to be evidence-based and independently verified in a transparent manner with appropriate analytical approaches [14, 15]. While frameworks have been proposed to evaluate wearables across the most commonly measured metrics, such as step count, heart rate, EE and VO_2_ max [13, 16], the majority of these relate to research-grade devices. To address the challenges discussed, the Towards Intelligent Health and Well-Being Network of Physical Activity Assessment (INTERLIVE) net was established to integrate and build upon previous work to propose best-practice protocols for the validation of exercise/activity metric measurement capabilities in consumer wearables and smartphones. In this case, INTERLIVE is addressing the measurement of EE. The network first conducted a systematic literature review to identify the methods previously used in the validation of EE estimation in consumer wearables and smartphones. Following this, other relevant literature discussing factors that may introduce bias in validation studies was then consulted, alongside evidence-informed network discussion to develop best-practice validation recommendations. This paper presents a comprehensive report on the variables to consider when designing and conducting validation protocols for the estimation of EE and makes recommendations for the best practice methodologies and reporting of such studies.

## Expert Statement Process

### The INTERLIVE Network

INTERLIVE is a joint initiative of the University of Lisbon (Portugal), German Sport University (Germany), University of Southern Denmark (Denmark), Norwegian School of Sport Sciences (Norway), University College Dublin (Ireland), University of Granada (Spain) and Huawei Technologies, Finland. The network was founded in 2019, comprising of the authors of this paper, plus additional experts within each institution, and strives towards developing best-practice protocols for evaluating the validity of consumer wearables with regard to measurement of exercise/activity metrics. Moreover, we are aiming to increase awareness of the advantages and limitations of different validation methods and to introduce novel health-related metrics, fostering a widespread use of physical activity indicators. To date, best-practice validation protocols for consumer wearable heart rate monitoring [17] and step-counting [18] have been proposed by the network. In this paper, we detail INTERLIVE’s work in respect of the measurement of EE.

### Expert Validation Protocol Development

#### Expert Validation Process

The network used the same process to develop the best-practice validation protocols for the consumer wearable device-derived metrics as was used for the previous HR monitoring and step-counting parameters [17, 18]. The first step for developing the expert recommendations consisted of a systematic review of the validation protocols used in the scientific literature. This information was then used as the foundation for discussions and to provide recommendations on the optimal and most feasible protocol for assessing the validity of consumer wearables. Working group meetings were held to discuss the aspects of the validation protocols used in the studies identified in the systematic search. A set of key domains for best-practice recommendations were proposed based on the outcomes of the systematic literature review, the a priori knowledge relating to research grade device validation [13, 16, 19, 20], and the evidence-informed expert opinion of the INTERLIVE members. The synthesised data were then reviewed with respect to these domains, and expert validation protocols for the consumer wearable-derived EE metric was then developed by the working group.

#### Systematic Review Process

The primary aim of the systematic literature review was to determine which methods and protocols are currently being used in the scientific literature to validate the consumer wearable or smartphone-derived metric of EE. It was beyond the scope of this review to explore the results of the included studies, although a recent systematic review and meta-analysis was published, with accuracy varying dependent on activity type [11]. The search was conducted with respect to the Preferred Reporting Items for Systematic Reviews and Meta-Analyses (PRISMA) statement and registered with the international database of prospectively registered systematic reviews in health and social care (PROSPERO ID: CRD42021223508). To identify peer-reviewed journal articles, we searched the PubMed, Web of Science, and Scopus electronic databases using specific search terms, which are listed in electronic supplementary Table 1.

To be included for review, the studies had to (1) be published prior to 4 December 2020; (2) examine the validity of consumer-based wearable and smartphone applications for estimating EE compared with the gold-standard criterion measure of either direct or indirect calorimetry, metabolic chamber, or DLW; and (3) be written in English. Studies were excluded if the index measure was not derived from data acquired using a consumer wearable device or smartphone, did not contain a validation protocol, there was no gold-standard criterion measure, the outcome assessed by the device was not EE, or if the full text was not available. For our purposes, wearables were restricted to only those with a consumer purpose rather than a research or clinical purpose, meaning that the devices are primarily marketed to the consumer and are readily available for purchase and use by members of the public. No exclusion criteria were set for the study population (i.e., healthy, clinical patients, children). The systematic literature review process was conducted by RA, MHR, JS, and JT. Title/abstract and full-text screening were completed by two independent reviewers and confirmed by a third independent reviewer using Covidence software (Veritas Health Innovation) [21].

A custom-designed spreadsheet was used for data extraction and included the following headings: sample size included/analysed, sex distribution, type of population, body mass index (BMI), height, weight, age, condition type (laboratory; semi-free-living; free-living), criterion measure (description, configuration and placement), index measure (description, configuration and placement), testing protocol, signal processing, data synchronisation, statistical analysis, results and conclusions. Validation protocols were divided into three categories based on the following definitions:

*Laboratory:* Well-controlled conditions, predominantly tasks such as walking or running on a treadmill/indoor/outdoor environment, or cycling on a stationary cycle ergometer at pre-defined or self-selected speeds.

*Semi free-living:* Semi-controlled conditions, including ‘simulated’ activities of daily living for the purpose of replicating ‘free-living’ conditions (e.g. sweeping, cooking, folding laundry, computer use, sport-specific activity).

*Free-living:* Uncontrolled methodologies that involve participants wearing the index device during ‘normal’ daily life, outside of a controlled laboratory or simulated environment.

## Current State of Knowledge

**Fig. 1:**
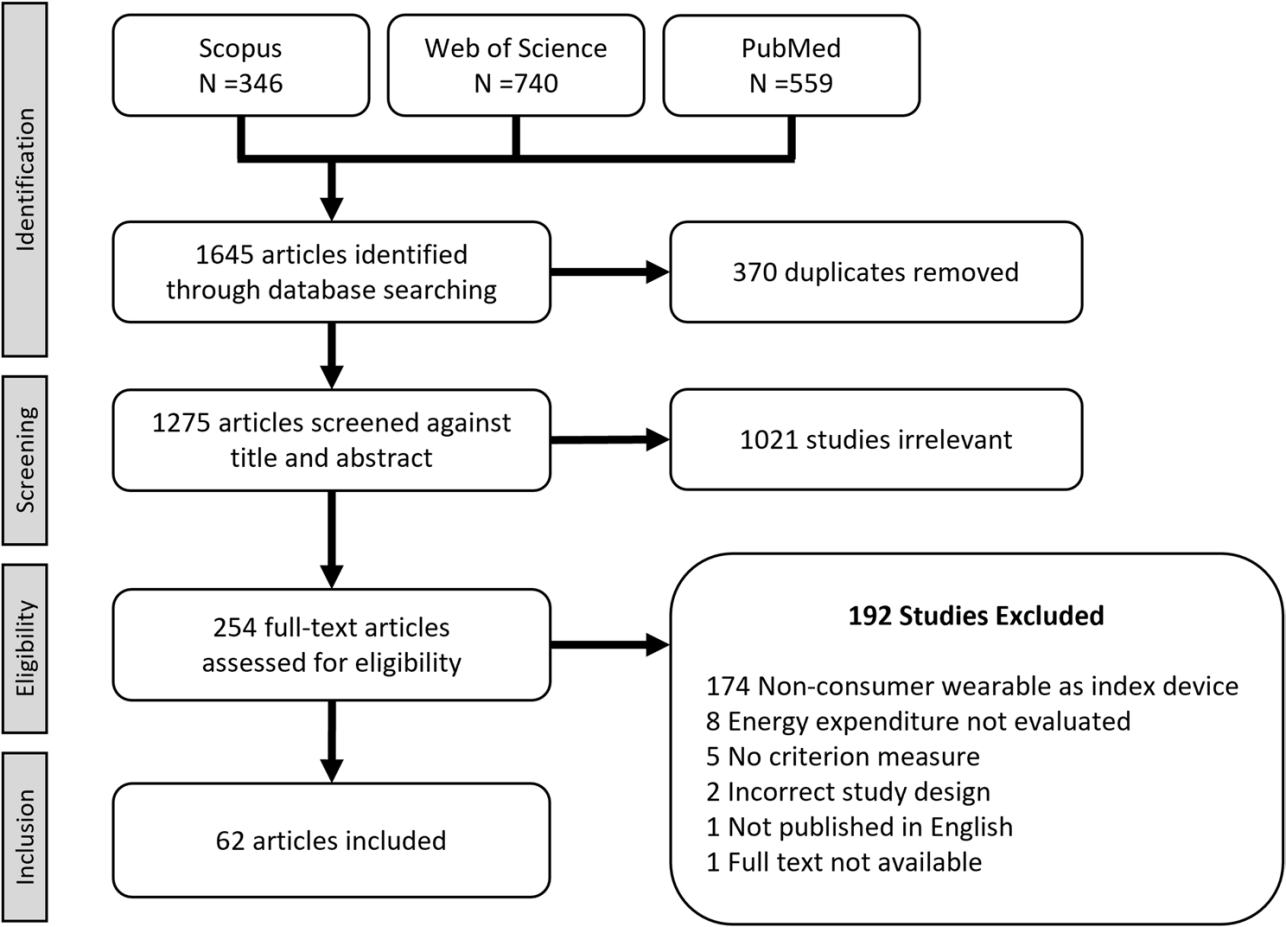
PRISMA flowchart of the systematic review process. PRISMA Preferred Reporting Items for Systematic Reviews and Meta-Analyses

The search strategy for this systematic review yielded 1645 potential articles for inclusion. Following the removal of 371 duplicates, 1275 titles and abstracts were screened, resulting in 254 articles that met the criteria for full-text screening. Overall, 192 studies were excluded, resulting in a final total of 62 studies accepted for data extraction. Of the 192 exclusions, 174 were excluded as they did not evaluate consumer wearables and instead assessed clinical and research-grade devices. The PRISMA flowchart of the stages of the systematic search and screening process, including reasons for exclusion, is illustrated in Fig. 1.

**Fig. 2:**
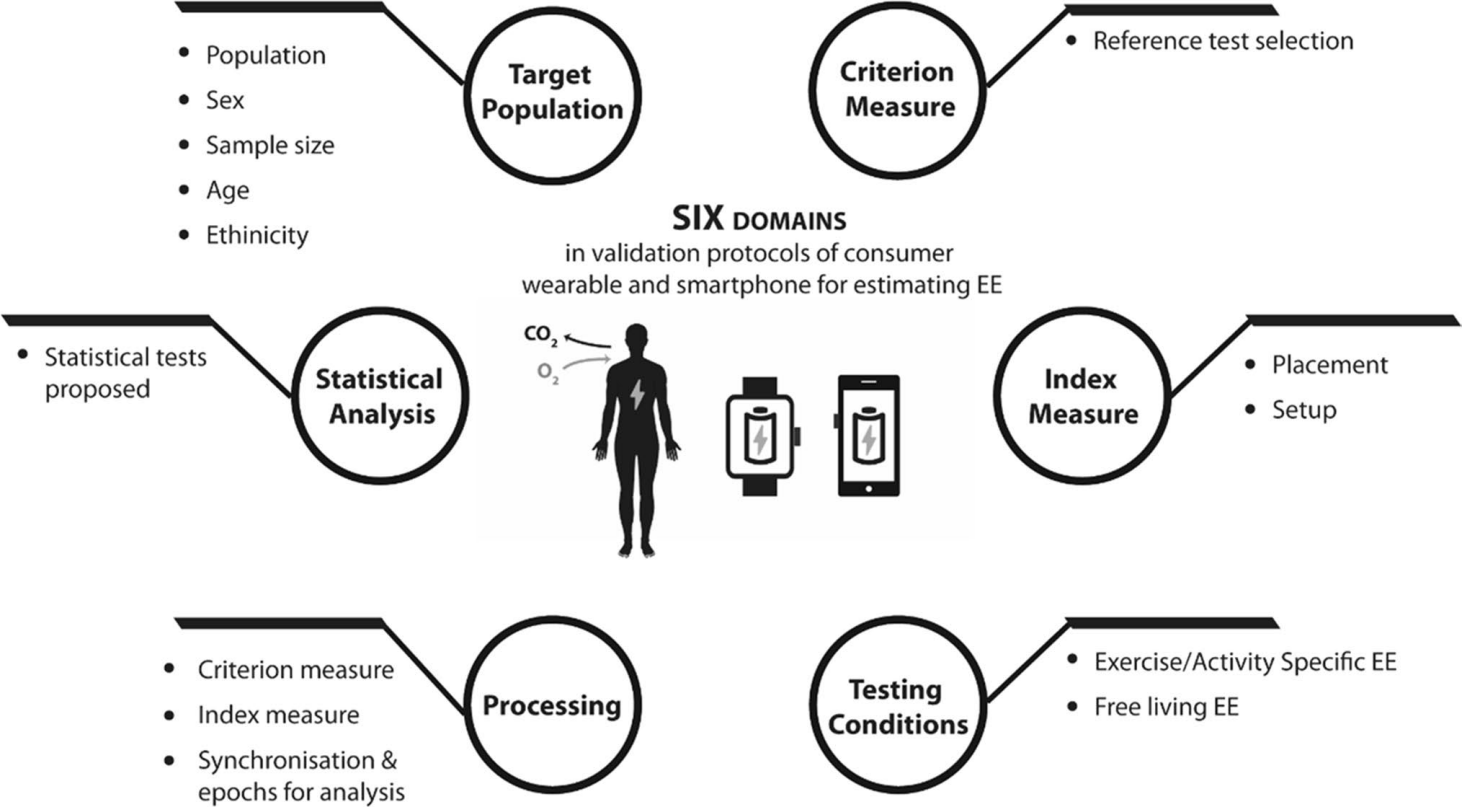
Six domains and corresponding variables of interest identified as being of importance in the validation of consumer wearable and smartphone estimation of EE. *EE* energy expenditure

The full extraction data presenting the methodologies employed across the included studies can be found in electronic supplementary Table 2 (laboratory protocols), electronic supplementary Table 3 (semi-free-living protocols), and electronic supplementary Table 4 (free-living protocols). Eighty-five different models from 35 manufacturers were reported in articles included in this systematic review, the majority being wearable manufacturers (*n* = 30), with only five smartphone applications evaluated. The most studied manufacturer was Fitbit, with at least one model evaluated in 33 articles, followed by the Apple Watch (*n* = 14), Garmin (*n* = 12), Jawbone (*n* = 11) and Polar (*n* = 10). Similar to the previous statements of the INTERLIVE network, the data are presented in six key validation protocol and reporting domains comprising of the target population, criterion measure, index measure, testing conditions, data processing and statistical analysis (Fig. 2). Within each domain, aspects deemed critical to effective validation and reporting were identified and are outlined with respect to the information collated during the statement process.

### Target Population

In the measurement of TEE, a number of variables contribute to individual variation in determining both resting metabolic rate (RMR) and AEE, including age, sex, body size and composition, ethnicity, genetics and fitness level [6, 13]. Consumer wearables are marketed to a broad global demographic, from adolescents to older adults as well as from athletes to those with sedentary lives. Thus, unless investigating a specific clinical application, validation studies of EE estimation should reflect the heterogenous nature of the target population in the sample, and not be confined to a niche sector. Twenty-seven (44%) of the studies identified in the systematic literature review recruited their sample from a university community. While this may be convenient, we recommend that if the aim of the study is validation of the wearable in the general population, then a more heterogenous sample is required with a wide variation in EE estimates. Notably, 13 of the 62 studies (21%) failed to provide any report of where the participants were sampled from, and 34 studies (55%) did not report how participants were recruited. This information should be a prerequisite in the reporting of any validation study.

RMR is well documented to decline with age [22–24], and while age is factored into many EE estimation equations [13], participants in validation studies should represent a spectrum of ages, with due consideration for stratification according to age ranges. The current literature tends to validate wearables in the younger adult population, with 35 studies reporting a sample with an average age in the 20 s. Older adults (> 60 years) are particularly poorly represented. Alongside a representative age group, it is important that validation protocols include an equal sample across both sexes, given the variance in metabolic rate [6, 25], and report on body size and composition. Finally, skin tone has previously been noted as having an effect on the accuracy of heart rate measures [17]. Given that many consumer wearables use heart rate in their EE estimation algorithm, if the device includes photoplethysmography, skin tone should also be reported using the Fitzpatrick scale [26].

The average sample size across all the studies in the systematic review was 34 participants (range 12–100), with only nine articles (15%) conducting a sample size calculation and justification. Despite the lack of sample size calculations, many studies conducted equivalence or difference tests, which are dependent on sample size. Therefore, if the objective of the study was to determine a minimum acceptable level of accuracy, a sample size calculation should be conducted based on pilot testing or previously published mean and standard deviation of the differences between the index and criterion measures [27]. If this is not the primary focus, for homogenous samples we recommend a minimum of 45 participants [28], although consideration should be given to the participant characteristics, and a larger sample size will likely be required for more heterogenous groups.

### Criterion Measure

It is widely regarded that the gold-standard approach to measuring TEE is by using DLW [6, 7, 29, 30]. This is assessed over a period of several days in a free-living context, and average daily TEE can be calculated over a specified time period via the collection of urine samples. In order to determine AEE, RMR is calculated via indirect calorimetry or prediction equations and then subtracted from the TEE measured with DLW [31]. However, it is not possible to measure activity type, intensity or duration within the TEE for each time period [32], and this information may be relevant for many users and clinicians. Equally, the use of DLW is expensive and requires in-depth specialist analytical facilities, which places a restriction on the feasibility of its deployment in many studies [30, 32]. Therefore, while the use of DLW is recommended in free-living protocols, other measures such as calorimetry are needed for the calculation of AEE in specific activities.

Calorimetry involves the measurement of either thermal change (direct calorimetry) or the measurement of gaseous exchange (indirect calorimetry), and can take the form of whole-room metabolic chambers, ventilated hoods, or a facemask/mouthpiece [33–35]. Indirect calorimetry is most commonly used to measure EE in the laboratory, as it is highly feasible to measure oxygen consumption and calculate energy cost, yet this is not practical in a free-living context [6]. The results of a recent systematic review and meta-analysis suggest that the accuracy of research-grade wearables for estimating EE is not at a satisfactory level for these devices to be used in the validation of commercial wearables as a criterion measure [11]. Therefore, based on the current available evidence, we recommend that the criterion measure for the validation of specific activities or exercise protocols be indirect calorimetry, with DLW used for free-living studies. All five studies in the systematic literature review validating consumer wearables in a free-living context utilised the DLW method [10, 36–39], while 3 (10%) of the 28 semi-free-living protocols utilised a metabolic chamber [10, 37, 40]. The remaining studies with a semi-free-living protocol (25) and all 52 laboratory protocols selected indirect calorimetry in the form of a portable system or metabolic cart. The criterion device should be placed according to manufacturer’s instructions and reported in the study write-up, alongside laboratory-specific data on the quality of the criterion measure where possible (i.e. percentage coefficient of variation [reliability]), and any calibration details. In the case of using DLW in free-living protocols and reporting on AEE, authors should provide a full account of assumptions and equations used [6], for example whether RMR is calculated via indirect calorimetry or by equation. We would recommend that the best-practice for measuring RMR in a free-living protocol is with indirect calorimetry, rather than estimated using equations; however, we recognise this may not always be feasible for researchers, and in those cases, the exact equation used should be stated.

### Index Measure

In order to ensure the ecological validity of a study protocol, the placement of the wearable device must be considered, especially in studies evaluating multiple devices at the same time. All commercial wearables should be evaluated with the device placed according to the manufacturer’s instructions. Activity monitors are commonly worn on the wrist, although in some cases may be attached to the waist of the user [41]. Less used placements include the torso [42] and the shoes [43]. Fifteen studies identified in the systematic review evaluated multiple wrist-worn devices on the same arm in the protocol, with up to three devices worn on one arm. It is unclear what effect this placement might have on the outputs of the device, and, arguably, while still placed on the lower forearm, at least one of the three devices is not being worn according to the manufacturer’s instructions. There is a negative impact to the feasibility of studies if researchers are required to only place one device on each wrist during a testing protocol, therefore we would recommend that where possible, researchers avoid placing multiple devices on the same wrist, with a maximum of two wrist-worn devices being tested on one arm simultaneously. In addition, when using two wrist-worn devices on the same arm, we recommend that there should be a random counterbalanced placement of the devices between participants; however, the order of device placement with respect to distance from the wrist joint should be clearly reported.

Equally, smartphone applications may not have an explicit manufacturer/developer-specified placement. Of the four studies in the systematic review that assessed smartphone measured EE, one placed the smartphone at the lower back [44], one on the belt next to a wearable at the mid-axillary line [45], one in a pocket (although the location of the pocket was not specified) [46], and the last study used two smartphones, one in the hand and one in the right pocket, with a comparison made between the two positions [47]. It has been reported that in day-to-day life, 60% of females place their phone in their bag, while 60% of males carry theirs in their pocket [48]. As such, we would consider clothing pockets, handbags or carrying in the hand as the most suitable positions to evaluate smartphone estimation of EE. The phone should not be mounted to the body in an unnatural way such as to the chest or low back.

As well as reporting the placement protocol in detail, the exact model and version of the index device should be clearly specified, for both the hardware and software, including noting if any firmware updates took place during the data capture period. Most studies reported the brand and device name (e.g. Fitbit Charge HR); however, with regular updates to hardware and software, it is difficult to fully replicate the study without specified version numbers. Furthermore, the device should be reset back to baseline between participants to ensure there is no influence on device EE algorithms potentially based on previous use. Finally, any demographic and anthropometric details inputted into the device, and the use of any specific exercise modes, should be reported. The use of exercise modes has been highlighted as an area for further examination, with researchers and users currently unaware of the impact of using different modes on the measurement of EE [49, 50].

### Testing Conditions

Consumer wearables are designed to provide a measure of EE in uncontrolled environments such as daily EE, with limited user interaction with the device, and during more controlled settings where EE is estimated during a specific exercise/activity. The results from the systematic review show that many researchers are favouring the use of laboratory or semi-free-living protocols. Of the 62 studies included in the systematic review, 51 (82%) included a laboratory protocol, 27 studies (44%) conducted some form of semi-free-living investigation, and only five studies (8%) examined free-living conditions.

Laboratory protocols seek to validate the wearable in measuring EE during tasks that are well controlled. The accuracy can be examined across varying intensities and include a combination of predefined walking, running, and cycling activities. The studies identified in the systematic literature search with laboratory protocols highlighted varying levels of accuracy of a wearable in measuring EE dependent on the intensity of the activity. Additionally, in the case of walking and running, the accuracy of the index measure varied dependent on the level of incline of the activities in a number of studies [42, 51–53]. In contrast, semi-free-living evaluation incorporates simulated activities of daily living and activities emulating sports or exercises that are more specific than walking, running or cycling. Within these protocols, participants are encouraged to complete the tasks as they would do during normal daily life, at a self-selected pace. The purpose of these type of validation studies is to capture (1) specific sports or complex activities, and/or (2) a diverse range of activities emulating activities of daily living, such as relaxation, housekeeping, or other physically active leisure pursuits. We argue that the accurate measurement of TEE and AEE during specific sports or complex activities is important because it allows end users to determine the validity of their device during activities of particular relevance to them. However, because of the constraints of laboratory equipment and the laboratory setting, many activities/sports, and particularly activities of daily living, cannot be performed in a manner that is comparable to the way these activities or exercise modes are performed on a daily basis. If this is not possible, we argue that the information acquired from the typical semi-free-living study has very little validity in determining the precision of EE from consumer devices during actual free-living behaviours of users. In contrast to our previous recommendations on the validation of consumer wearables to measure heart and step count [17, 18], we recommend that validation of TEE and AEE from wearables should be performed within two testing conditions: (1) during specific activities or exercises/sports, and (2) in a free-living context.

In order to accurately assess EE during a specific exercise or activity, a distinction between steady state and variable intensity activities must be made. During steady-state activities, steady state must be achieved where heart rate remains stable during a continuous task and cardiac output is at a sufficient level to support oxygen transport in order to meet energy cost [54–56], before measurements are included in the analysis. There is some debate in the literature about when steady state is achieved, but the general consensus is that there is a 2- to 5-min period where *V*O_2_ and *V*CO_2_ varies by up to 15% from the EE of the performed work, depending on the activity and the change in intensity gradient [55–59]. Therefore, we recommend validation of steady-state activities be performed across different intensities to determine validity at different absolute levels, with researchers providing justification of the achievement of steady state. As a general rule of thumb, but dependent on the intensity gradient and the absolute intensity performed, each of the activities/intensities within an exercise/activity-specific protocol is recommended to last at least 6 min to facilitate stable steady-state measurements. We recommend conducting evaluation with as wide a range of intensities as feasible and a consideration of the relevance of graded activities (e.g. an incline ≥ 5% during walking or running tasks). Equally, if conducting walking/running assessment on a treadmill, we would recommend all testing is conducted with a 1% incline to account for the reduced metabolic cost of treadmill propulsion [60]. Due consideration should be given to the potential need for a recovery period between activity bouts when higher-intensity exercises are examined to allow sufficient recovery to approximately RMR. The choice of all intensities should take into consideration the characteristics of the sample, with calculated intensities based on the participant’s maximal heart rate or *V*O_2max_ being the most preferable over selecting absolute values, or lesser still, self-selected intensities. However, while these personalised intensities are recommended, there may be cases where it is not feasible to calculate personalised fitness levels, and, in these instances, conducting and reporting absolute exercise intensities such as speed or watts is recommended.

During these activities, consideration of available workout modes on devices are needed as the mode selected dictates what sensors are enabled on the device in order to preserve battery, for example indoor activities disable the GPS and cycling does not require step detection, and these modifications are likely to impact the validity of the measurements. These user-defined activities for assessment of EE tend to relate to the most common workouts, including, but not limited to, running, walking, cycling and swimming. Attempting to validate devices in other tasks that are not defined by the manufacturer, such as reading, vacuuming or sweeping, is most appropriately assessed in the validity of TEE estimation in the free-living environment. Additionally, a further issue that must be addressed is the need to correct for inaccuracies in the measurement of EE at heavy to maximal intensity activity, where the anaerobic component will not be captured by indirect calorimetry. Researchers conducting high-intensity activities should recognise this, adjust for it using an appropriate method such as incorporating blood lactate measurements [61, 62] or interpolation based on exercise intensity [63], and report these methods appropriately.

To assess EE estimation of consumer wearables in the real-world, free-living protocols should capture a range of activities from participants in uncontrolled conditions. Given the recommendation for the use of DLW in free-living validation, the protocol period should last 7–14 days, and participants should not be constrained in their activities in any way [30, 64]. The reporting of such protocols should include in detail the instructions given to participants, including whether they were asked to use or did use any specific exercise modes on the wearables during the period. Finally, it is preferable to record and report on the adherence of participants to wearing the device as instructed to highlight any discrepancies between devices due to non-adherence, and report on any missing data. In the case of specific activity EE estimation, it is recommended that data must be captured with the participant wearing the device for the entirety of the protocol, and if there is non-adherence to this or there are missing data, then that data capture be discarded. Furthermore, for free-living evaluations, any methods used to interpolate missing data, such as during charging overnight, should be reported clearly.

### Processing

The data processing and reporting that is provided in validation studies is important but is often overlooked, with numerous articles in the systematic review leaving the reader left to make assumptions on the processing methods of the study. Authors must provide clear detail on how steady-state or anaerobic EE (if relevant) was managed in their protocol, and how the data collection, including synchronisation, was managed to capture the measures of the criterion and index device per activity. Given the 2- to 5-min period that is required to achieve steady state, we would recommend that the average of the last 3 min of the exercise protocols for steady-state exercises or activities be used to calculate EE.

There is variance in the reporting of the type of EE being measured, with lack of clarity in papers regarding whether the measure is TEE or AEE, and in the units used, with kilocalories per minute (kcals/min), kilocalories per activity (kcals/activity) and metabolic equivalents (METs) all being described in the literature. Recent literature reports MET minutes to be the unit of choice for EE validation [15], yet while the time-phased nature of these units are desirable for comparability, we do not feel that the consumer market is currently able to understand MET minutes. Furthermore, most consumer devices report EE in kcals. As such we recommend that the preferred metric should be kcals per unit of time (minute/day). Furthermore, researchers should specify which equation is used to determine EE from gaseous consumption, report all related assumptions, and are clear in reporting whether the study assessed TEE or AEE. Equally with regard to the index measure, as much information as possible should be provided on the inputs to the EE calculation of the device. Reporting that a proprietary algorithm was used should be deemed as the minimum criteria, with attempts made to note any of the inputs to that specific EE algorithm, including heart rate, accelerometer data, user demographics and subjective reporting such as rate of perceived exertion. This is somewhat challenging in many cases, given the ‘black-box’ nature of these proprietary algorithms that manufacturers do not wish to divulge. Additionally, where possible, researchers are encouraged to follow open science principles in making raw data available using appropriate data repositories to facilitate combined analyses in the future.

Finally, the management of synchronisation between devices is another potential source of error. The approach to this will vary depending on the study design and devices used. As such, researchers must be cognisant of data synchronisation when designing the study, and the synchronisation process undertaken should be described in as much detail as possible in the report to allow for adequate replication.

### Statistical Analysis

When assessing the validity of an index device to a criterion measure, the levels of accuracy of the index device should be calculated with agreement between the two devices [65, 66]. The Bland–Altman approach to limits of agreement has become widely used in the current medical literature [67], including 38 studies (61%) in the systematic review conducting this approach in the analysis. However, there was large variance in the statistical approaches used, with many studies utilising measurements of correlation (e.g. Pearson, Spearman) [*n* = 38, 61%], comparison of means (e.g. *t* test) [*n* = 22, 36%], and measures of relative reliability (e.g. intraclass correlation coefficient) [*n* = 13, 21%]. These approaches have been highlighted as inappropriate when assessing agreement between a measurement tool and the reference standard in medical instrument validation studies [65].

To comprehensively assess the validity of the index device in estimating EE, we recommend the use of Bland–Altman limits of agreement analysis in combination with mean absolute percentage error (MAPE). The Bland–Altman method provides a measure of the agreement between the criterion and index device; researchers should state if the assumptions for valid limits of agreement analysis were fulfilled [67], and, in addition, should incorporate least-products regression to assess for proportional or fixed bias, as described by Ludbrook [66]. The use of MAPE allows for comparison between devices and testing conditions and is the average of the absolute error of a tool. It is commonly used in describing the error of a prediction [68], and 29 studies (47%) included in our systematic review used this approach in their analysis. The use of both methods and related visualisations provides a comprehensive assessment of the group- and individual-level validity of the consumer device in estimating EE and will illustrate any bias occurring.

To allow readers to assess the validity of a device in estimating EE, it is important for the contextual use of the device to be considered. For example, a much greater level of accuracy would be required when applying the measure in a clinical trial setting compared with EE monitoring in a general wellness application. Regardless of the context, the level of error, limits of agreement and 95% confidence intervals should be reported clearly according to the validation conditions, with an avoidance of binary hypothesis testing, particularly when a sample size calculation has not been conducted.

### Recommended Validation Protocol

Based on the current state of knowledge and the evidence described within this statement, INTERLIVE recommends that manufacturers of consumer wearables and smartphones that provide an estimate of EE utilise standardised validation methodologies and report this in a transparent and replicable manner. Studies should be designed to validate devices across both specific sports/activities (focusing on the predefined exercise modes offered by the device, if any) and in uncontrolled environments. This evaluation should be conducted using the appropriate criterion measure with a relevant sample, in conditions that best reflect the expected use of the device.

These recommendations aim to ensure that many of the sources of bias identified during the review of the literature are addressed. Table 1 presents a detailed best-practice validation protocol and reporting requirements, while Table 2 presents a checklist of items to be considered during validation protocol planning.

**Table 1:** Proposed best-practice protocols for the validation of wearable and smartphone-derived energy expenditure

Domain	Variable	Protocol consideration	Reporting consideration
Target population	Population	If the purpose is to validate wearable-derived EE for the general healthy population, a broad heterogeneous sample should be used If the purpose is to use the wearable in a specific clinical application, validation should be performed in an homogenous sample	Report the target population used and method of recruitment
	Age	When assessing validity for the general healthy population, participants should be representative of a specific group from children/adolescents, adults, or older adults	Average and range of sample age should be reported
	Sex	Include an equal sample of males and females within the study	The number of female and male participants should be reported Report on measures of body size and composition (i.e. weight, height, BMI)
	Ethnicity	If HR is contributing to EE estimation, ethnicity and skin tone should be reported * If technology used by the device to estimate EE is unknown, then ethnicity and skin tone should be reported	Report ethnicity and race of the study population Report skin tone using the Fitzpatrick scale
	Sample size	A sample size calculation should be completed based on the mean and standard error of the differences between device and criterion from either previously published or pilot study data If previous data are not available, we recommend at least 45 participants	A sample size justification should be provided The sample size recruited and the sample size analysed should be clearly reported
Criterion measure	Reference test selection	*Exercise/activity-specific EE:* Direct or indirect calorimetry *Free-living EE:* DLW	Report the criterion measure used, calibration criteria, and laboratory-specific %CV For DLW, specifics on isotope dosing, collections and analysis method should be reported
Index device	Placement	*Wearable activity monitors* should be worn on the body in accordance with the manufacturer’s instructions. If wrist worn, a maximum of 2 devices per wrist should be used at the same time, with placement being randomly counterbalanced between participants *Smartphones* should be hand-held or placed in the clothing pocket, handbags or purses, belt phone holder, or any other position that would typically be used in daily living. Non-typical placement such as strapping the phone to the chest or mounting to the body in an unnatural way should be avoided	Report the placement of the device and information on the order of placement if more than one wrist-worn device is used Report on distance from the wrist
	Set-up	All demographic details required by the device for participant initiation should be inputted If the device has the option to select a specific exercise mode (i.e. indoor running, cycling, walking, etc.), choose the mode that best reflects the activity being performed Device should be reset back to baseline/factory settings between each participant to ensure there is no influence on algorithms based on historical use	Report the device model and version and whether firmware updates took place during data acquisition Report what demographic details are imputed into the device per participant for initiation Report what mode (if any) is used during each activity (i.e. indoor running, cycling, walking, etc.) If individual calibration is required, ensure this is reported in detail
Testing conditions	Exercise/activity-specific EE	Activities should be chosen based on the predefined activity modes available on the index device Each work-bout should last at least 6 min. Due consideration should be given to the potential need for a recovery period between activity bouts when higher-intensity exercises are examined to allow sufficient recovery to approximately RMR Consideration should be given to establishing steady state within each activity The protocol should include a wide-range of intensity zones: Walking—at least three intensities plus one at incline > 5% Running—at least two intensities plus one at incline > 5% * If this takes place on a treadmill, then all activities should include a 1–2% incline to account for the metabolic cost of treadmill propulsion Cycling—at least three intensities The intensities and activities used should take into consideration the characteristics and capabilities of the sample, with calculated intensities based on the participant’s maximal HR or VO_2max_ being the preferable option over selecting absolute values or self-selected intensities. When not feasible to calculate personalised fitness levels, reporting absolute exercise intensities (i.e. watts, speed, rpm, etc.) are recommended over participant self-selected intensities	Report the type of activity performed and equipment used (i.e. treadmill running, cycle ergometer, etc.). For each activity, include information on the duration, inclination and intensities used, including how intensity was determined (HR/VO_2max_, absolute values, or self-selected pace) Report on if and how steady state was established Report on if and how anaerobic EE was measured when exercising at higher intensities
	Free-living EE	The free-living protocol should involve a duration of 7–14 days whereby the participant completes activities of daily living in an unconstrained environment (home, work, travelling, etc.)	Describe the duration of the free-living protocol and the instructions given to the participants during this time Specify whether participants were asked to use specific modes during the period Report on the adherence of participants to wearing the index device as instructed Report methods of interpolation of missing data in free-living evaluations
Processing	Criterion	*Exercise/activity-specific EE:* During steady-state activities, ensure measurements captured before steady state are not included in the analysis At higher/maximal intensities, appropriate methods should be utilised to adjust the criterion measurement to incorporate anaerobic EE *Free-living EE:* DLW will provide the average TEE over a 7- to 14-day period. To calculate AEE, RMR must be determined using either indirect or direct calorimetry (preferred method) or validated prediction equations	The equations used to determine EE from gaseous consumption and related assumptions should be provided For calculating AEE from TEE derived from DLW, clarify how RMR was determined (i.e. estimated from direct/indirect calorimetry or equation) in addition to any other assumptions or equations used Results should be reported as kilocalories per minute Where possible, raw data should be made available in an appropriate repository in line with open science principles
	Index	If available, it is preferable to use minute-by-minute data for calculating AEE from the device If minute-by-minute data are not available, calculate EE at steady state for each activity; note EE once steady state is reached (pre-EE) and then again post activity. Subtract the post-EE from the pre-EE and divide by the number of minutes between pre- to post-EE to get kilocalories per minute	Report on how the device displays EE (i.e. AEE, TEE) Results should be reported as kilocalories per minute If available, describe the algorithm and inputs used by the device (i.e. HR, accelerometry, GPS, demographics, etc.) to derive EE. If no information is available, it should be reported that EE was derived using a proprietary algorithm
	Synchronisation and epochs for analysis	When possible, use demarcation events for marking timestamps of achieving steady state and end of activity for both criterion and index measure to facilitate synchronisation	Provide as much detail as possible on the synchronisation process in order to allow for study replication
Statistical analysis	Statistical tests	To assess device accuracy, the following statistical tests should be performed per activity/category: 1. Bland–Altman with limits of agreement 2. Least products regression of the differences against the means 3. MAPE Subgroup analysis is encouraged if sample size allows (e.g. sex, age category, ethnicity, BMI, etc.)	Include an illustration of the Bland–Altman plots (a supplementary file may be needed if multiple index devices are tested) Binary conclusions about the validity of the device should not be made if a formal sample size analysis has not been conducted

*EE* energy expenditure, *DLW* doubly labelled water, *TEE* total energy expenditure, *AEE* activity energy expenditure, *HR* heart rate, *RMR* resting metabolic rate, *VO*_*2max*_ maximal oxygen consumption, *BMI* body mass index, *%CV* percentage coefficient of variation, *MAPE* mean absolute percentage error

**Table 2:** Checklist of items to be considered during the validation protocol of wearable and smartphones to estimate energy expenditure

**Target population assessment**
Age
Children (< 12 years)
Adolescents (12–18 years)
Adults (18–65 years)
Older adults (> 65 years)
Sex (equal sample of males and females)
Sample size Calculated based on previously published or pilot study data OR If previous data are not available, at least 45 participants
**Criterion measure assessment**
*Exercise/activity specific testing* Direct or indirect calorimetry *Free-living testing* DLW
Placement of criterion according to the manufacturer’s instructions (applies only to indirect calorimetry)
**Index device assessment**
*Placement:* Wearable activity monitors placed according to the manufacturer’s instructions Smartphones either handheld or placed in places typically used in everyday living (i.e. pocket, handbags, belt phone holder)
*Device setup:* All demographic details required by the device are inputted Specific exercise mode chosen if applicable
**Exercise/activity specific EE assessment**
Walking, running, and/or cycling with three different intensities and one inclination > 5%
Intensity based on either participant maximal HR or VO_2max_, absolute value, or participant self-selected pace
At least 6-min duration for each activity bout with ample recovery time allotted in-between activity bouts performed at higher intensities
**Free-living EE assessment**
Participant wears index device for 7–14 days while simultaneously undergoing a DLW protocol
**Processing**
Criterion measure processing
*Exercise/activity EE:* The average of the last 3 min of the exercise used to calculate steady-state EE
*Free-living EE:* RMR determined using either indirect or direct calorimetry or validated prediction equations
Index measure processing
Minute-by-minute data for calculating AEE from the device used if available If minute-by-minute data are not available, pre-EE (EE at the start of steady state for each activity) is subtracted from post-EE (EE post activity) and divided by the number of minutes between pre- to post-EE to get kilocalories per minute
Index and criterion synchronisation
**Statistical analysis**
Bland–Altman with limits of agreement per activity/category
Least products regression of the differences against the means
MAPE

*DLW* doubly labelled water, *HR* heart rate, *EE* energy expenditure, *AEE* activity energy expenditure, *RMR* resting metabolic rate, *VO*_*2max*_ maximal oxygen consumption, *MAPE* mean absolute percentage error

## Discussion and Future Directions

This INTERLIVE network expert statement aims to provide clear and actionable recommendations and guidelines for the comprehensive and replicable evaluation of the validity of consumer wearables in estimating EE. In consultation with the evidence base, wider literature and expert knowledge, a detailed validation protocol is described covering domains of the target population, criterion measure, index measure, validation conditions (exercise/activity-specific or free-living), processing and statistical analysis.

The lack of both a regulatory standard for the validation process of consumer wearables and an acceptable context-specific level of accuracy facilitates the heterogeneity of methodologies used in the validation of EE estimation seen in this systematic literature review. This can lead to all stakeholders experiencing difficulties in comparing various devices and ultimately deciding which might be more suitable or appropriate for a specified purpose. A standardised approach to validation would benefit all stakeholders and ensure a transparent and objective method of validation, which will ultimately drive the sector towards more accurate, and therefore more useful, devices. This would benefit (1) consumers, facilitating them to be able to make an informed choice on the most suitable device or application; (2) healthcare providers, by being better placed to adopt such devices/applications as part of their digital health strategy; and (3) manufacturers and developers, who can clearly illustrate the value of their products.

This transparency of reporting opens an interesting debate, as the ‘black-box’ nature of EE estimation algorithms means that manufacturers do not wish to divulge the proprietary information that constitutes these models. However, in order to make sufficient comparisons and maximise the accuracy of the device, we mirror calls in the literature for companies to be more open about the algorithms that they employ [12]. While respecting the proprietary nature of these devices, we recommend that as a minimum, manufacturers and researchers should declare the key components that act as inputs for the EE estimation algorithm. For example, despite a device being equipped with an accelerometer, GPS, and photoplethysmography sensor, this does not automatically mean that all of these sensors are utilised as inputs into the EE model, and additional metrics such as demographics and anthropometrics that are also included should be stated. Depending on the inputs declared, we would draw readers’ attention and consideration to the previous INTERLIVE statements for the validation of heart rate [17] or step counting [18] in consumer wearables to determine whether these inputs into the EE algorithm are valid.

This systematic review highlighted a lack of studies validating EE from wearable devices during anaerobic conditions or during very high intensities (above lactate threshold). This leaves a gap in current validation studies as strength training, conditioning exercises and high-intensity interval training (HIIT) are growing in popularity [69]. Indirect calorimetry has clear limitations when used to determine EE during resistance training [70], with anaerobic and recovering EE reported to be significantly larger than the aerobic component of EE during a single set of training to fatigue [61]. As such, this may require the use of additional criterion measures such as blood lactate measurement, together with modification to the indirect calorimetry timing, to capture excess post-exercise oxygen consumption [61]. Further research to identify an acceptable standard of measurement of anaerobic EE and variable intensity, non-steady-state activity is required in order to make suitable recommendations for optimal protocols for assessing the accuracy of wearables in estimating EE in those conditions [71].

It is important not to understate the challenges in implementing EE validation studies. The notable lack of free-living validation, which is an important step for many consumer users, is likely due to issues in feasibility, given the longitudinal nature of these protocols, the resources required for DLW, and the additional need to measure RMR. Nonetheless, there is currently a lack of evidence regarding the validity of consumer wearables in the measurement of AEE in daily life. One research group that has conducted validation in the free-living context showed close correlation between consumer devices and DLW for TEE; however, when assessing the estimation of AEE across a 15-day period, a far greater number of devices demonstrated significant differences to the criterion measure [10, 37]. This would suggest that the accuracy for consumer devices to measure TEE is driven by the standard RMR formulas incorporating user characteristics such as age, sex, height and weight, rather than the sensors on board the devices. Therefore, further validation of the measurement of AEE in free-living conditions is warranted to truly understand the value such devices can offer in EE estimation.

Additionally, these protocol recommendations do not include evaluation of reliability for activity-specific EE estimation, yet reliability is a prerequisite for validity. Therefore, researchers may want to consider the various aspects of reliability in their protocols depending on the intended use-case. This may include repeating a particular testing condition/intensity during the same session when the participant has recovered to RMR, or to repeat the protocol on an alternate day. Equally, many index device manufacturers do not provide exercise/activity modes for sport-specific activity, including soccer and basketball. This is potentially due to poor performance of these devices in estimating EE during such activities, but presents an interesting opportunity for manufacturers to develop exercise modes for popular recreational sports, which would make them more appealing to consumers. For these modes to be useful, their validation needs to mimic a range of actual sports-specific behaviours, and not only running with or kicking a ball.

## Conclusions

This INTERLIVE expert statement provides an evidence-informed best-practice protocol for the validation of consumer wearables in estimating EE. The systematic literature review conducted as part of the formation of this statement highlighted a heterogeneity between methodologies in key domains, particularly in the target populations, data processing and statistical approaches, giving rise to validation bias. Additionally, the lack of free-living validation leads to limited abilities of users to understand the accuracy of the device in its intended use case. The INTERLIVE network recommends that the proposed validation protocol is used when considering the validation of any consumer wearable or smartphone measure of EE to provide a robust validation of the device. Adherence to this validation standard will help ensure methodological and reporting consistency, facilitating comparisons between consumer devices and the amalgamation of standardised open datasets. This will ensure that manufacturers, consumers, healthcare providers and researchers can use this technology safely and to its full potential.

## Supplementary Information

Below is the link to the electronic supplementary material.

Supplementary file1 (DOCX 145 kb)

## Declarations

### Data availability

All data generated or analysed during this study are included in this published article (and its supplementary information files).
