# Supplementary material for: Recommendations for Determining the Validity of Consumer Wearables and Smartphones for the Estimation of Energy Expenditure: Expert Statement and Checklist of the INTERLIVE Network
Source: Sports Med. 2022 Mar 9;52(8):1817–32. doi: 10.1007/s40279-022-01665-4 (PMC9325806; doi:10.1007/s40279-022-01665-4)
Supplement: Supplementary file 1 — Supplementary file1 (DOCX 145 kb) [file 40279_2022_1665_MOESM1_ESM.docx]

## Supplemental File

# Recommendations for determining the validity of consumer wearables and smartphones for the estimation of energy expenditure: expert statement and checklist of the INTERLIVE network.

Rob Argent^1,2,3^, Megan Hetherington-Rauth^4^, Julie Stang^5^, Jakob Tarp^5^, Francisco B Ortega^6,7^, Pablo Molina-Garcia^6^, Moritz Schumann^8,9^, Wilhelm Bloch^8^, Sulin Cheng^8,9^, Anders Grøntved^10^, Jan Christian Brønd^10^, Ulf Ekelund^5^, Luis B Sardinha^4^, Brian Caulfield^1,2^

1. Insight Centre for Data Analytics, University College Dublin, Ireland.
2. School of Public Health, Physiotherapy and Sport Science, University College Dublin, Ireland.
3. School of Pharmacy and Biomolecular Sciences, Royal College of Surgeons in Ireland, Dublin, Ireland.
4. Exercise and Health Laboratory, CIPER, Faculdade de Motricidade Humana, Universidade de Lisboa, Lisboa, Portugal.
5. Department of Sport Medicine, Norwegian School of Sport Sciences, Oslo, Norway.
6. PROFITH (PROmoting FITness and Health through physical activity) Research Group, Department of Physical Education and Sports, Faculty of Sport Sciences, University of Granada, Spain.
7. Department of Bioscience and Nutrition, Karolinska Instutet, Sweden.
8. Institute of Cardiovascular Research and Sports Medicine, Department of Molecular and Cellular Sports Medicine, German Sport University, Cologne, Germany.
9. Exercise Translational Medicine Centre, the Key Laboratory of Systems Biomedicine, Ministry of Education, and Exercise, Health and Technology Centre, Department of Physical Education, Shanghai Jiao Tong University, Shanghai, China.
10. Department of Sports Science and Clinical Biomechanics, Research Unit for Exercise Epidemiology, Centre of Research in Childhood Health, University of Southern Denmark, Odense M, Denmark.

Corresponding author:
Dr Rob Argent, rob.argent@insight-centre.org

*Supplemental Table 1: Search terms used in Embase, PubMed, and Web of Science databases excluding study design.*

| **Scopus** | **Web of Science** | **Pubmed** |
| --- | --- | --- |
| **Index device**  TITLE-ABS-KEY ( "wearable*"  OR  "smartwatch*"  OR  "smart watch*"  OR  "smartphone*" OR "smart phone*"  OR  "watch*"  OR  smartband*  OR  "smart band*"  OR  smartbracelet*  OR  "smart bracelet*"  OR "activity monitor*" OR "activity track*" ) | **Index device**  ALL=( wearable*  OR  smartwatch*  OR  "smart watch*"  OR  smartphone* OR "smart phone*"  OR  watch*  OR  smartband*  OR  "smart band*"  OR  smartbracelet*  OR  "smart bracelet*"  OR "activit* monitor*" OR "activit* track*" ) | **Index device**  ( "Wearable Electronic Devices" [Mesh] OR "Fitness Trackers" [Mesh] OR "wearable*" OR "smartwatch*" OR “smart watch” or “smartwatches” OR "smart watch*" OR  "smartphone*" OR "smart phone*" OR "watch*" OR “smartband*”  OR "smart band*" OR  smartbracelet* OR "smart bracelet*" OR "activity monitor*" OR "activity track*" ) |
| **Outcome**  TITLE-ABS-KEY ( "energy expenditure"  OR  "energy consumption"  OR  "calori*"  OR  "calori* expenditure"  OR  "activ* energy"  OR  "energy metabolism"  OR  "energy cost"  OR  "energy balance" ) | **Outcome**  ALL= ( "energ* expenditure"  OR  "energ* consumption"  OR  "calori*"  OR  "calori* expenditure"  OR  "activ* energ*"  OR  "energ* metabolism"  OR  "energ* cost"  OR  "energ* balance" ) | **Outcome**   ( "Energy Metabolism" [Mesh] OR "energy expenditure"  OR  "energy consumption"  OR  "calori*" OR "calori* expenditure"  OR  "activ* energy"  OR  "energy metabolism"  OR  "energy cost"  OR  "energy balance" ) |
|  |  |  |
| **Study Design**  TITLE-ABS-KEY ("reproducibility of results" OR "valid*" OR "validity" OR "'validation" OR "validate" OR "comparison" OR "reliab*" "reliability" OR "reliable") | **Study Design**  ALL= ("reproducibility of results" OR valid* OR comparison OR reliab* ) | **Study Design**  ("Reproducibility of Results" [Mesh] OR "valid*" OR "validity" OR "'validation" OR "validate" OR "comparison" OR "reliab*" "reliability" OR "reliable") |
| **N studies included: 346** | **N studies included: 740** | **N studies included: 559** |

Date: 07/12/20 04/12/20 04/12/20

*Supplemental Table 2: Studies using Laboratory Protocols. ICC; intraclass correlation coefficient, MAPE; mean absolute percent error, RMSE; root mean square error, CV; coefficient of variation.*

| **Author (year)** | **Target population &**  **Recruitment** | **N**  **age (SD) or range**  **%females** | **Types of activities** | **Duration/**  **repetitions** | **Index device (placement)** | **Specified inputs to index device** | **Criterion** | **Statistical comparison** |
| --- | --- | --- | --- | --- | --- | --- | --- | --- |
| Adam Noah et al. (2013) [1] | Healthy adults  Convenience sample from University community | n= 23  27 (8) yrs  43% | Treadmill walking, treadmill inclined walking, jogging and stair stepping | 6 mins/activity | Fitbit (waist)  Fitbit Ultra (waist) | ‘subject parameters’ - not further declared. | Indirect calorimetry | ICC, comparison of means |
| Alsubheen et al. (2016) [2] | Healthy adults  Convenience sample from University community | n= 13  40 (12) yrs  38% | Treadmill walking,  treadmill inclined walking | 10 mins/activity | Garmin Vivofit (wrist) | none declared | Indirect calorimetry | ANOVA, comparison of means |
| Bai et al. (2016) [3] | Healthy adults  Convenience sample from University and surrounding community | n= 52  18 – 65 yrs  46% | Treadmill walking or jogging | 25 mins | Fitbit Flex (wrist)  Polar Loop (wrist)  Misfit Shine (wrist)  Nike+ Fuelband SE (wrist)  Jawbone JU24 (wrist) | none declared | Indirect calorimetry | Bland-Altman plot, MAPE, correlation, equivalence testing, mean bias |
| Bai et al. (2018) [4] | Healthy adults  Convenience sample from University and surrounding community | n= 39  32 (11) yrs  41% | Treadmill walking or jogging | 25 mins | Apple Watch 1 (wrist)  Fitbit Charge HR (wrist) | none declared | Indirect calorimetry | Bland-Altman plots, MAPE, correlation, equivalence testing, mean percent error, RMSE |
| Bassett et al. (2000) [5] | Healthy adults  Convenience sample from University and surrounding community | n= 81  40 (15) yrs  53% | Treadmill walking w/wo carrying load | 15 mins/activity. 12 participants for each activity. | Yamax SW701 (waist) | Body mass and stride length | Indirect calorimetry | Bland-Altman plot, correlation, t-tests, error score |
| Boudreaux et al. (2018) [6] | Healthy adults  Convenience sample, not further declared | n= 50  22 (3) yrs  51% | Ergometer cycling | 2 mins/stage | Apple Watch Series 2 (wrist)  Fitbit Blaze (wrist)  Fitbit Charge 2 (wrist)  Polar PH7 (chest)  Polar PA360 (wrist)  Garmin Vivosmart HR (wrist)  TomTom Touch (wrist) | Sex, age, height, and weight | Indirect calorimetry | ICC, MAPE, t-test |
| Chowdhury et al. (2017) [7] | Healthy adults  Not declared | n= 30  27 (6) yrs  50% | Treadmill walking, treadmill jogging, ergometer cycling, climbing stairs | 10 mins/activity, stairs for 5 mins | Apple Watch (wrist)  Microsoft Band (wrist)  Fitbit Charge HR (wrist)  Jawbone UP24 (wrist) | none declared | Indirect calorimetry | Bland-Altman plot, mean absolute error, mean percent error, RMSE, Pearson correlation, ANOVA, equivalence testing |
| Compagnat et al. (2019) [8] | Stroke patients  Convenience sample from a physical medicine and rehabilitation department | n= 26  65 (interquartile range: 56 – 77) yrs  not declared | Walking | 6 mins | Geonaute ONStep 400 pedometer (non-paretic hip) | none declared | Indirect calorimetry | Bland-Altman plot, RMSE, correlation |
| Dannecker et al. (2013) [9] | Healthy adults  Convenience sample from two communities | n= 19  27 (7) yrs  47% | Treadmill walking, ergometer cycling, stepping | 10 mins/activity | Phillips DirectLife (hip)  Fitbit Tracker (hip) | Fitbit: annotation of activities via web-based software | Indirect calorimetry | RMSE, percent RMSE, ANOVA, mean standard error |
| Diaz et al. (2015) [10] | Healthy adults  Convenience sample from University community | n= 24  range: 20 – 51 yrs  54% | Treadmill walking, treadmill jogging | 6 mins/activity | Fitbit One (hip)  Fitbit Flex (wrist) | none declared | Indirect calorimetry | Correlation, mean difference, percent bias |
| Diaz et al. (2016) [11] | Healthy adults  Convenience sample from University community | n= 13  32 (9) yrs  100% | Treadmill walking, treadmill jogging | 6 mins/activity | Fitbit One (hip)  Fitbit Flex (wrist) | none declared | Indirect calorimetry | Bland-Altman plot, relative percent error, agreement, t-test |
| Dooley et al. (2017) [12] | Healthy adults  Convenience sample from University community | n= 62  23 (4) yrs  58% | Treadmill walking, treadmill jogging | 4 mins/activity | Apple Watch (wrist)  Fitbit Charge HR (wrist)  Garmin Forerunner 225 (wrist) | none declared | Indirect calorimetry | Bland-Altman plot, MAPE, correlation, ANOVA, Cohen's d effect size. |
| Düking et al. (2020) [13] | Healthy adults  Not declared | n= 25  26 (7) yrs  56% | Treadmill walking, treadmill running, sprints including directional changes on a SpeedCourt | 5 mins/activity, sprint to completion | Apple Watch Series 4, version 5.1) (wrist)  Polar Vantage V, firmware 3.1.7 (wrist)  Garmin Fenix 5, software 7.6 (wrist)  Fitbit Versa, version 32.33.1.30 (wrist) | Apple, Polar, and Garmin: relevant 'activity modes' selected on the | Indirect calorimetry | Standardized mean bias, typical error, CV, correlation |
| Falter et al. (2019) [14] | Cardiovascular disease  All patients scheduled for a cardiopulmonary exercise test as part of their cardiopulmonary rehabilitation | n= 40  62 (15) yrs  20% | Ergometer cycling | to exhaustion | Apple Watch Sport 42 mm, first generation (wrist) | Cycling mode | Indirect calorimetry | Bland-Altman plot, MAPE, ICC, mean difference, MAE |
| Gilgen-Ammann et al. (2019) [15] | Healthy adults  Convenience sample of inactive, active and endurance athletes. Not further declared. | n= 30  30 (5) yrs  50% | Indoor walking and running on 290meter track, ergometer cycling | time to complete | Polar Vantage M (wrist) | Relevant 'modes' (e.g. running) selected. Participant’s anthropometrics, resting HR, HRmax, and VO2max values | Indirect calorimetry | Bland-Altman plot, MAPE, correlation, equivalence test, absolute percent error, mean absolute error with regression |
| Godino et al. (2020) [16] | Children  Convenience sample from class announcements and email lists | n= 60  10 (1) yrs  52% | Treadmill walking, treadmill walking with backpack, ergometer cycling, climbing stairs, outdoor walking | 5-6 mins/activity or to completion of course | Fitbit Charge HR (wrist) | none declared | Indirect calorimetry | Bland-Altman plot, MAPE, t-test, regression, |
| Hendrikx et al. (2017) [17] | Healthy adults  Not declared | n= 29  41 (14) yrs  52% | Treadmill walking, treadmill included walking, ergometer cycling, cross-trainer, walking, running, cycling | 3 mins/activity | Phillips Health Watch (wrist) | none declared | Indirect calorimetry | Bland-Altman plot, MAPE, equivalence test, mean absolute error |
| Herkert et al. (2019) [18] | Coronary artery disease or heart failure patients  Convenience sampling from outpatient clinics, randomly selected from a list of patients participating in previous studies | n= 38  63 (7) yrs  18% | Treadmill walking, cycle ergometer | 3 mins/activity | Fitbit Charge 2 (wrist)  Mio Slice (wrist) | Mio Slice: 'Workout' mode activated  Fitbit Charge: sex, age, height, and weight | Indirect calorimetry | Bland-Altman plot, ICC, RMSE, t-test |
| Hopkins et al. (2020) [19] | Healthy adults  Not declared | n= 12  29 (4) yrs  0% | Ergometer cycling | To exhaustion | VitalScout, VitalScout app v2.0.6 (chest) | none declared | Indirect calorimetry | Correlation |
| Imboden et al. (2018) [20] | Healthy adults  Not declared | n= 30  49 (19) yrs  50% | Walking, running, treadmill walking, treadmill running | 2 – 15 mins | Fitbit Flex (wrist)  Fitbit One (waist)  Fitbit Zip (waist)  Jawbone UP24 (wrist) | Sex, height, weight and age. | Indirect calorimetry | Bland-Altman plot, MAPE, percent bias, correlation, ANOV, /t-test |
| Johnson et al. (2016) [21] | Healthy adults  Convenience sample from University community | n= 29  22 (2) yrs  52% | Walking at indoor track, treadmill running | 15 mins/activity | 2x Samsung Galaxy S4, S Health app, version 2.0.0.009 (hand and pocket) | Sex, age, height, weight, and 'activity mode' set to 'moderate activity level' and applying the 'pedometer' option | Indirect calorimetry | Bland-Altman plot, correlation, ANOVA |
| Kane et al. (2010) [22] | Healthy adults  Recreational or competitive runners. Not further declared | n= 20  24 (4) yrs  45% | Treadmill walking, treadmill inclined walking, treadmill running | 6 mins/activity | Nike+ (shoe) | Individual calibration performed per manufacturer’s instructions (400 self-paced walk + run) | Indirect calorimetry | ANOVA |
| Kendall et al. (2019) [23] | Healthy adults  Convenience sample from University community | n= 50  26 (8) yrs  50% | Treadmill walking, treadmill running with inclination | To exhaustion | Polar FT7 (chest)  Basis B1 (wrist)  Fitbit Flex (wrist)  Omron HJ-321 (hip)  Jawbone UP Move (hip) | Polar FT7: age, weight, height, gender, maximum HR, training intensity.  Basis B1, Fitbit Flex, Jawbone UP: age, height, weight, gender.  Omron HJ-321: weight and height. | Indirect calorimetry | ICC, mean difference, t-test |
| LaMunion et al. (2020) [24] | Children and adolescents  Convenience sample from community | n= 100 (89 with any data)  12 (3) yrs  51% | Overground walking, overground running ergometer cycling | All performed twice, for 60-90 seconds and for 4-5 mins | Devices were not worn on manufacturer specified locations due to the number of devices being examined  Apple Watch 2 (wrist)  Mymo Activity Tracker (waist)  Misfit Shine 2 (waist and shoe)  Fitbit Charge 2 (wrist)  Samsung Gearfit (wrist) | Not specifically specified beyond: ‘All device profiles were updated before each trial using the participant’s demographic information.’ | Indirect calorimetry | Bland-Altman plot, MAPE, t-test, ANOVA |
| Lee et al. (2014) [25] | Healthy adults  Convenience sample from University and surrounding community | n= 60  26 (6) yrs  50% | Treadmill walking, treadmill running, overground walking, overground walking with 15 kg backpack | Treadmill activities for 3 mins, others for 5 mins | DirectLife (chest)  Fitbit One (waist)  Fitbit Zip (waist)  Jawbone UP Band (wrist)  NikeFuel Band (wrist)  Basis B1 Band (wrist)  Bodymedia Fit (arm) | Devices were initialized with age, sex, height, weight, handedness, smoker/non-smoker | Indirect calorimetry | Bland-Altman plot, MAPE, equivalence test, correlation, RMSE |
| Leenders et al. (2003) [26] | Healthy adults  Not declared | n= 28  24 (4) yrs  61% | Treadmill walking | 30 mins/activity | Yamax-Digiwalker-500 (hip) | Body mass | Indirect calorimetry | Bland-Altman plot, ANOVA, correlation |
| Lemmens et al. (2018) [27] | Pregnant women (12-35 weeks pregnant)  Women referred through primary care. Not further declared | n= 51  31 (5) yrs  100% | Walking indoors walking outdoors, ergometer cycling, cross trainer | 1-3 mins/activity, cycling for 6 minutes | Optical Heart Rate Monitor based on technology in Phillips Cardio and Motion monitoring Module, CM3-Generation-1 (wrist) | none declared | Indirect calorimetry | Bland-Altman plot, equivalence test, RMSE, mean bias |
| Machač et al. (2013) [28] | Diabetes type 1 and 2  Not declared | n= 19  57 (8) yrs  68% | Treadmill walking, treadmill inclined walking | 15 mins/activity | Omron HJ-720 (hip) | Step length and body mass | Indirect calorimetry | Correlation, percent bias |
| Maddison et al. (2017) [29] | Healthy adults  Convenience sample from University community | n= 21  27 (8) yrs  62% | Treadmill walking, treadmill running | 5 mins/activity | Moto G Smartphone using Movn App, Android version 4.3 (hip) | none declared | Indirect calorimetry | Bland-Altman plot, correlation, mean difference |
| Montoye et al. (2017) [30] | Healthy adults  Not declared | n= 32  24 (1) yrs  44% | Treadmill walking, treadmill running, treadmill inclined walking | 5 mins/activity | Fitbit Charge HR (wrist)  Hexoskin (torso) | Sex, age, height and weight | Indirect calorimetry | Bland-Altman plot, MAPE, correlation, ANOVA/t-test |
| Moreno et al. (2020) [31] | Healthy adults + wheelchair users  Convenience sample from University community | n= 30  36 (14) yrs  40% | Wheel-chair treadmill, arm cycle ergometry | 3 mins/activity | AppleWatch Series 1 (wrist) | Mode set to ‘wheelchair’ | Indirect calorimetry | Bland-Altman plot, MAPE, ICC, mean difference, t-test |
| Nolan et al. (2016) [32] | Healthy adults  Convenience sample from University community | n= 25  25 (3) yrs  56% | Treadmill walking, treadmill running | 30 mins/activity | iPod Touch / iPhone with bespoke app (lower back) | 3-axial 'feature extraction' using data driven approach. METs estimated from 'acceleration features', walk/running speed, height, weight and sex | Indirect calorimetry | Bland-Altman plot |
| Nuss et al. (2019) [33] | Healthy adults  Not declared | n= 30  24 (3) yrs  50% | Treadmill walking, treadmill running | To exhaustion | Apple Watch (wrist)  Fitbit Charge HR 2 (wrist) | Sex, age, height and weight | Indirect calorimetry | MAPE, correlation, t-test, mean difference |
| O'Driscoll et al. (2020a) [34] | Healthy adults  Weight Loss Maintenance study and convenience sample from Community | n= 59  44 (14) yrs  69% | Treadmill walking, treadmill running, ergometer cycling | 5 mins/activity | Fitbit Charge 2 (wrist) | Sex, age, height and weight | Indirect calorimetry | Bland-Altman plot, MAPE, correlation, RMSE, mean difference, equivalence test, mean absolute error, absolute percent error |
| O'Driscoll et al. (2020b) [35] | Healthy adults  Weight Loss Maintenance study and convenience sample from Community | n= 59  44 (14) yrs  69% | Treadmill walking, treadmill running, ergometer cycling | 5 mins/activity | Fitbit Charge 2 (wrist) | 2 bespoke algorithms based on; 1) Fitbit HR above sitting HR, steps, age, gender, height, weight, and 2) 1 + fat mass and fat-free mass | Indirect calorimetry | MAPE, RMSE, correlation, equivalence testing |
| Parak et al. (2017) [36] | Healthy adults  Not declared | n= 24  36 (8) yrs  46% | Treadmill running | To exhaustion | PulseOn (wrist) | 'Neural network' used to derive VO2 which was converted to EE using information from the calorimeter. The 'neural network' included information on height, weight, gender, age and HR during a submaximal running test. | Indirect calorimetry | Bland-Altman plot, MAPE, mean absolute error, correlation |
| Passler et al. (2019) [37] | Healthy adults  Not declared | n= 24  23 (2) yrs  46% | Treadmill running | 10 mins/activity | Garmin Vivosmart HR (wrist)  TomTom Touch (wrist)  Withings Pulse Ox (wrist) | Garmin Vivosmart HR: 'Anthropometric data' and activity log activated  TomTom Touch: sex, weight, training intensity Withings Pulse Ox: 'Anthropometric data'. Additionally, examined with and without adding perceived effort of activity | Indirect calorimetry | Bland-Altman plot, MAPE, mean absolute error, ICC, t-test |
| Price et al. (2017) [38] | Healthy adults  Convenience sample from University community | n= 14  23 (6) yrs  79% | Treadmill walking, treadmill running | 4 mins/activity | Fitbit One (hip)  Garmin Vivofit (wrist)  Jawbone UP (wrist) | Participant details entered in device-specific web accounts | Indirect calorimetry | Bland-Altman plot, Correlation, t-test, MAPE, relative error |
| Reddy et al. (2018) [39] | Healthy adults  Convenience sample from University community | n= 20  28 (6) yrs  55% | Treadmill walking, treadmill running, ergometer cycling | To exhaustion | Garmin Vivosmart HR+ (wrist)  Fitbit Charge 2 (wrist) | Appropriate exercise modes used (running or cycling) | Indirect calorimetry | Bland-Altman plot, correlation, t-test, ANOVA, mean relative error, MAPE |
| Roos et al. (2017) [40] | Healthy adults  Convenience sample of recreational or competitive runners. Not further declared. | n= 20  24 (2) yrs  40% | Treadmill running | Lower intensity (≤70% VO2peak) stages performed for 10 mins, stages at 90% and 110% VO2peak performed for 90 seconds | Suunto Ambit2  Garmin Forerunner920XT  Polar V800  Participants wore all three watches at the same time. Two watches on the left wrist and forearm, the third watch on the right wrist, and the heart rate monitors around the chest. The positioning of the watches and the localization of the heart rate monitors was randomized. | Sex (Polar V800 only), age, height, weight, HRmax.  The training profile “running” and for Garmin Forerunner920XT “indoor running” was selected from each watch’s menu. | Indirect calorimetry | Bland-Altman plot, correlation, t-test, ANOVA, mean relative error, MAPE |
| Sasaki et al. (2015) [41] | Healthy adults  Convenience sample from University community | n= 20  24 (5) yrs  50% | Treadmill walking, treadmill jogging, treadmill inclined walking | 6 mins/activity | Fitbit Classic (waist) | Sex, age, height and weight | Indirect calorimetry | Bland-Altman plot, correlation, t-test, mean percent difference |
| Shcherbina et al. (2017) [42] | Healthy adults  Convenience sample from University community | n= 60  38 (11) yrs  52% | Treadmill walking, treadmill running, ergometer cycling | 5 mins/activity | AppleWatch (wrist)  Basis Peak (wrist)  Fitbit Surge (wrist)  Microsoft Band (wrist)  MIO Alpha 2 (wrist)  PulseOn (wrist)  Samsung Gear S2 (wrist) | none declared | Indirect calorimetry | Bland-Altman plot, principal component analysis |
| Slootmaker et al. (2009) [43] | Healthy adults  Convenience sample from University community | n= 32  29 (7) yrs  56% | Treadmill walking, stair stepping | 5 mins/activity | PAM model AM101 (waist) | None declared | Indirect calorimetry | Generalized estimation equation, ICC, CV |
| Swartz et al. (2009) [44] | Healthy adults  Not declared | n= 48  33 (11) yrs  58% | Treadmill walking | 5 mins/activity | Omron HJ-700IT (waist)  Sportbrain iStep X1 (waist) | Omron: body mass and stride length  Sportbrain: sex, height, weight, age, stride length | Indirect calorimetry | Bland-Altman plot, ANOVA |
| Thiebaud et al. (2018) [45] | Healthy adults  Recreationally active adults. Not further declared | n= 22  22 (3) yrs  9% | Treadmill walking, treadmill running | 3 mins/activity | Fitbit Surges (wrist)  Microsoft Band (wrist)  Tomtom cardios (wrist) | Treadmill mode | Indirect calorimetry | Bland-Altman plot, correlation, equivalence testing, MAPE |
| Wahl et al. (2017) [46] | Healthy adults  Convenience sample from University community | n= 20  25 (3) yrs  50% | Treadmill walking, treadmill running, running outdoors | 5 mins/activity. Running outdoors time to complete 2.4 km | Bodymedia Sensewear MF (arm)  Polar Loop (wrist)  Beurer AS80 (wrist)  Fitbit Charge (wrist)  Fitbit Charge HR (wrist)  Garmin Vivofit (wrist)  Garmin Vivosmart (wrist)  Garmin Vivoactive (wrist)  Garmin Forerunner 920XT (wrist)  Withings Pulse Ox (arm) | Sex, height, weight, age, body fat, handedness | Indirect calorimetry | Bland-Altman plot, MAPE, ICC, typical error |
| Wallen et al. (2016) [47] | Healthy adults  Convenience sample from University community | n= 22  25 (6) yrs  50% | Treadmill walking, treadmill running, ergometer cycling | walking/running: 3 mins/activity. Cycling: 18 mins | Apple Watch (arm)  Fitbit Charge HR (arm)  Samsung Gear S (arm)  Mio Alpha (arm) | Sex, age and ‘anthropometrical data’ | Indirect calorimetry | Bland-Altman plot, correlation, ICC, regression |
| Xie et al. (2018) [48] | Healthy adults  Convenience sample from University community | n= 44  22.3 (2.1) yrs  48% | Outdoor walking, outdoor running, outdoor cycling | walking on a 400m track for 2 laps.  running 1 lap.  Cycle 3 trips back and forth in a predetermined route | Sequentially from the elbow to the hand: Apple Watch 2,  Samsung Gear S3,  Fitbit Surge,  Jawbone Up3,  Xiaomi Mi Band 2,  Huawei Talk Band B3.  Dongdong, App (pocket)  Ledongli, App (pocket) | None declared | Indirect calorimetry | MAPE, correlation, t-test |
| Yavelberg et al. (2018)[49] | Healthy adults  + Type 1 diabetes  Not declared | n= 5 – 7  25 (8) yrs  Not declared | Treadmill walking or running, ergometer cycling | Total of 40 minutes | Garmin Vivofit2 (wrist)  Mio Fuse (wrist) | None declared | Indirect calorimetry | T-test |
| Zhang et al. (2019a) [50] | Healthy adults  Convenience sample from University community | n= 30  20 (2) yrs  43% | Treadmill walking, treadmill running | 10 mins/activity | Apple Watch Series 1 (wrist)  Fitbit HR (wrist)  LifeTrak (wrist) | Apple Watch: participant’s information such as sex, height, and weight entered before the exercise protocol  Fitbit HR: synced to an iPhone to obtain their physical characteristics  LifeTrak: did not require synchronization with smart phones for individual bio information input. | Indirect calorimetry | Bland-Altman plot, correlation, MAPE, equivalence testing |
| Zhang et al. (2019b)[51] | Healthy adults  Convenience sample from University community | n= 30  20 (2) yrs  43% | Treadmill walking, treadmill running | 10 mins/activity | Apple Watch (wrist) | None declared | Indirect calorimetry | ANOVA, correlation, coefficient of determination, ICC |

*Supplemental Table 3: Studies using Semifree-Living Protocols. ICC; intraclass correlation coefficient, MAPE; mean absolute percent error, RMSE; root mean square error, CV; coefficient of variation.*

| **Author (year)** | **Target population & Recruitment** | **N**  **age (SD) or range**  **females/males** | **Types of activities** | **Duration/**  **repetitions** | **Index device (placement)** | **Specified inputs to index device** | **Criterion** | **Statistical comparison** |
| --- | --- | --- | --- | --- | --- | --- | --- | --- |
| Alsubheen et al. (2016) [2] | Healthy adults  Convenience sample from University community | n= 13  40.0 (11.9) yrs  38% | Sedentary activities  (computer work, reading articles and writing) | 1 hr | Garmin Vivofit (wrist) | Proprietary algorithm | Indirect calorimetry | ANOVA, comparison of mean |
| Bai et al. (2016) [3] | Healthy adults  Convenience sample from University and surrounding community | n= 52  18 – 65 yrs  46% | Sedentary activities  (e.g. computer work, reading, writing – any activities requiring sitting without speaking).  Resistance exercises  (self-selected sets, repetitions and weights on any of 12 TechnoGym training equipment) | 20 mins on sedentary activities, 25 mins on resistance exercises activities | Fitbit Flex (wrist)  Polar Loop (wrist)  Misfit Shine (wrist)  Nike+ Fuelband SE (wrist)  Jawbone JU24 (wrist) | none declared | Indirect calorimetry | Bland-Altman plot, MAPE, correlation, equivalence testing, mean bias |
| Bai et al. (2018) [4] | Healthy adults  Convenience sample from University and surrounding community | n= 39  32 (11) yrs  41% | Sedentary activities  (seated at a desk using laptop, read book or use phone).  Activities of daily living  (folding laundry, sweeping, moving light boxes, stretching, slow walking) | 20 mins on sedentary activities, 25 mins on free-living activities | Apple Watch 1 (wrist)  Fitbit Charge HR (wrist) | none declared | Indirect calorimetry | Bland-Altman plots, MAPE, correlation, equivalence testing, mean percent error, RMSE |
| Bassett et al. (2000) [5] | Healthy adults  Convenience sample from University and surrounding community | n= 81  40 (15) yrs  53% | 1 to 9 activities from 6 broad categories of;  Yardwork, occupation, housework, family care, conditioning, recreation | 15 mins/activity. 12 for each activity | Yamax SW701 (waist) | Body mass and stride length | Indirect calorimetry | Bland-Altman plot, correlation, t-tests, error score |
| Boudreaux et al. (2018) [6] | Healthy adults  Convenience sample. Not further declared | n= 50  22 (3) yrs  56% | Resistance exercises  (chest press, latissimus dorsi pulldown, leg extension, leg curl) | 2 mins/stage | Apple Watch Series 2 (wrist)  Fitbit Blaze (wrist)  Fitbit Charge 2 (wrist)  Polar PH7 (chest)  Polar PA360 (wrist)  Garmin Vivosmart HR (wrist)  TomTom Touch (wrist) | Sex, age, height, and weight | Indirect calorimetry | ICC, MAPE, t-test |
| Brugniaux et al. (2010) [52] | Healthy adults  Convenience sample of regular hikers | n= 31  44 (7) yrs  52% | 9.7 km hike in France carrying a 4-5 kg backpack Total ascent and descent of 130 meters | ≈ 5 km/h | Polar AW200 (wrist) | Activity, altitude changes, weight, height, age, sex | Indirect calorimetry (performed in only 5 participants - criterion calculated for remaining sample) | Bland-Altman plot, correlation, regression, t-test, mean bias |
| Chowdhury et al. (2017) [7] | Healthy adults  Not declared | n= 30  27 (6) yrs  50% | Sedentary activities  (seated typing on laptop).  Activities of daily living  (simulated loading and unloading of dishwasher, sweeping) | 5 mins/activity, | Apple Watch (wrist) Microsoft Band (wrist)  Fitbit Charge HR (wrist)  Jawbone UP24 (wrist) | none declared | Indirect calorimetry | Bland-Altman plot, mean absolute error, mean percent error, RMSE, correlation, ANOVA, equivalence testing |
| Dannecker et al. (2013) [9] | Healthy adults  Convenience sample from two communities | n= 19  27 (7) yrs  47% | Sedentary activities  (watching tv, lying on bed, computer work).  Activities of daily living  (sweeping, standing) | 20 mins/activity | Phillips DirectLife (hip)  Fitbit Tracker (hip) | Fitbit: annotation of activities via web-based software | Indirect calorimetry | RMSE, percent RMSE, ANOVA, mean standard error |
| Gilgen-Ammann et al. (2019) [15] | Healthy adults  Convenience sample of inactive, active and endurance athletes. Not further declared | n= 30  30 (5) yrs  50% | Sedentary activities  (seated reading).  Activities of daily living  (wiping the floor, hanging out laundry, self-controlled strength training, ballgames (individually)) | 10 mins/activity | Polar Vantage M (wrist) | Relevant 'modes' (e.g. running) selected. Participants anthropometrics, resting HR, HRmax, and VO2max values | Indirect calorimetry | Bland-Altman plot, MAPE, correlation, equivalence test, absolute percent error, mean absolute error with regression |
| Godino et al. (2020) [16] | Children  Convenience sample from class announcements and email lists | n= 60  10 (1) yrs  52% | Sedentary activities  (sitting quietly, listening to music, playing games).  Activities of daily living  (agility drills) | 5 mins/activity | Fitbit Charge HR (wrist) | none declared | Indirect calorimetry | Bland-Altman plot, MAPE, t-test, regression, |
| Hendrikx et al. (2017) [17] | Healthy adults  Not declared | n= 29  41 (14) yrs  52% | Sedentary activities  (watching TV, desk work, lying down).  Activities of daily living  (washing dishes, folding towels, vacuum cleaning, standing) | Rest: 15 mins, other activities: 3 mins/activity | Phillips Health Watch (wrist) | none declared | Indirect calorimetry | Bland-Altman plot, MAPE, equivalence test, MAE |
| Herkert et al. (2019) [18] | Coronary artery disease or heart failure  Convenience sampling from outpatient clinics, randomly selected from a list of patients participating in previous studies | n= 38  63 (7) yrs  18% | Sedentary activities  (sitting, typing).  Activities of daily living  (standing, table cleaning, unloading dishwasher, climbing stairs) | 10 mins/activity | Fitbit Charge 2 (wrist)  Mio Slice (wrist) | Mio Slice: 'Workout' mode activated  Fitbit Charge: sex, age, height, and weight | Indirect calorimetry | Bland-Altman plot, ICC, RMSE, t-test |
| Imboden et al. (2018) [20] | Healthy adults  Not declared | n= 30  49 (19) yrs  50% | Sedentary activities  (lying, watching tv, computer use, writing, reading, playing cards).  Activities of daily living  (standing, dusting, sweeping, vacuuming, folding laundry, making bed, picking up items, gardening) | 2 – 15 mins/activity | Fitbit Flex (wrist)  Fitbit One (waist)  Fitbit Zip (waist)  Jawbone UP24 (wrist) | Sex, height, weight and age. | Indirect calorimetry | Bland-Altman plot, MAPE, percent bias, correlation, ANOVA/t-test |
| LaMunion et al. (2020) [24] | Children and adolescents  Convenience sample from community | n= 100 (89 with any data)  12 (3) yrs  51% | Sedentary activities  (supine rest, sitting, using the internet, reading a book, playing computer games).  Activities of daily living  (sweeping, dusting, playing catch with a football, self-paced stair climbing, playing soccer,  playing basketball, self-paced jumping jacks) | Participants selected ≥12 of 21 activities. Duration was  self-determined at 2-15 minutes. At least 40 mins had to be  sedentary | Devices were not worn on manufacturer specified locations due to the amount of devices being examined  Apple Watch 2 (wrist)  Mymo Activity Tracker (waist)  Misfit Shine 2 (waist and shoe)  Fitbit Charge 2 (wrist)  Samsung Gearfit (wrist) | Not specifically specified beyond: ‘All device profiles were updated before each trial using the participant’s demographic information.’ | Indirect calorimetry | Bland-Altman plot, MAPE, t-test, ANOVA |
| Lee et al. (2014) [25] | Healthy adults  Convenience sample from University and surrounding community | n= 60  26 (6) yrs  50% | Sedentary activities  (reclining, computer writing).  Activities of daily living  (stairs, stationary bike, elliptical exercise, wii, basketball) | 5 mins/activity | DirectLife (chest)  Fitbit One (waist)  Fitbit Zip (waist)  Jawbone UP Band (wrist)  NikeFuel Band (wrist)  Basis B1 Band (wrist)  Bodymedia Fit (arm) | Devices were initialized with age, sex, height, weight, handedness, smoker/non-smoker | Indirect calorimetry | Bland-Altman plot, MAPE, equivalence test, correlation, RMSE |
| Lemmens et al. (2018) [27] | Pregnant women (12-35 weeks pregnant)  Women referred through primary care. Not further declared | n= 51  31 (5) yrs  100% | Sedentary activities  (sitting, desk work).  Activities of daily living  (stacking groceries, vacuuming, folding towels, cooking or washing dishes, cleaning table, yoga, stairs, outdoor cycling) | 1-3 mins/activity | Optical Heart Rate Monitor based on technology in Phillips Cardio and Motion monitoring Module, CM3-Generation-1 (wrist) | none declared | Indirect calorimetry | Bland-Altman plot, equivalence test, RMSE, mean bias |
| Morris et al. (2019) [53] | Healthy adults  Convenience sample from University and surrounding community | n= 47  28 (11) yrs  53% | Resistance exercises  (body weight squats, push-ups, sit-ups, body weight lunges, pull-ups, running in place, high-knees, box step-ups) | 15 mins, 12 repetitions per exercise, as many exercises as possible.  N for each activity ranges from 10 to 30. | Fitbit Charge (wrist)  Fitbit One (hip)  Nike Fuel Band (wrist)  Jawbone UP Move (hip) | none declared | Indirect calorimetry | Correlation, MAPE, ANOVA, equivalence test, ICC |
| Murakami et al. (2016) [54] | Healthy adults  Not declared | n= 21  32 (10) yrs  57% | Simulated day consisting of eating, watching TV, using a computer, cleaning, walking on treadmill, sleeping | 24 hrs | Fitbit Flex (wrist)  Jawbone UP24 (wrist)  Misfit Shine (wrist)  Epson Pulsence PS-100 (wrist)  Garmin Vivofit (wrist)  TANITA AM-160 (pocket)  OMRON CaloriScan HJA-403C (pocket)  Withings Pulse O2 (waist) | none declared | Whole-room indirect calorimetry | Correlation, mean difference |
| Murakami et al. (2019) [55] | Healthy adults  Not declared | n= 21  32 (10) yrs  57% | Simulated day consisting of eating, watching TV, using a computer, cleaning, walking on treadmill, sleeping | 24 hrs | Fitbit Flex (wrist)  Jawbone UP24 (wrist)  Misfit Shine (wrist)  Epson Pulsence PS-100 (wrist)  Garmin Vivofit (wrist)  TANITA AM-160 (pocket)  OMRON CaloriScan HJA-403C (pocket)  Withings Pulse O2 (waist) | none declared | Whole-room indirect calorimetry | MAPE, correlation, mean difference |
| Nelson et al. (2016) [56] | Healthy adults  Convenience sample from University | n= 30  49 (19) yrs  50% | Sedentary activities  (lying down, watching TV, writing, reading, playing cards, using computer).  Activities of daily living  (standing, dusting, sweeping, vacuuming, laundry, making bed, picking up from floor, gardening) | Lying: 10 min; all others: 5 mins/activity | Fitbit One (hip)  Fitbit Zip (hip)  Fitbit Flex (wrist)  Jawbone UP24 (wrist) | Age, sex, height, weight | Indirect calorimetry | MAPE, mean absolute error, RMSE |
| O'Driscoll et al. (2020a) [34] | Healthy adults  Weight Loss Maintenance study and convenience sample from Community | n= 59  44 (14) yrs  69% | Activities of daily living  (‘folding’, sweeping) | 5 mins/activity | Fitbit Charge 2 (wrist) | Sex, age, height and weight | Indirect calorimetry | Bland-Altman plot, MAPE, correlation, RMSE, mean difference, equivalence test, mean absolute error, absolute percent error |
| Reddy et al. (2018) [39] | Healthy adults  Convenience sample from University community | n= 20  28 (6) yrs  55% | Sedentary activities  (sitting/lying)  Activities of daily living  (washing dishes, loading/unloading dishwasher, sweeping or vacuuming, organizing a room, scrubbing wall, carpet, and floor, climbing stairs).  Resistance exercises  (dumbbell bicep curls, Romanian deadlifts, Bulgarian split squat, dumbbell bench press, dumbbell shoulder press, and dumbbell step ups, 2 sets of 8 repetitions) | 3 mins/activity. Resistance exercise to completion | Garmin Vivosmart HR+ (wrist)  Fitbit Charge 2 (wrist) | Appropriate exercise modes used (running or cycling) | Indirect calorimetry | Bland-Altman plot, correlation, t-test, ANOVA, mean relative error, MAPE |
| Reeve et al. (2013) [57] | Healthy adults  Convenience sample from University community | n= 18  23 (5) yrs  39% | Resistance exercises  (squat, bench press, lunge, bent-over row, shoulder press, calf raise, bicep curl, triceps extension and abdominal curl) | 10 repetitions, 3 sets, 70% estimated 1RM | BodyMedia FIT (arm) | not declared | Indirect calorimetry | Correlation, percent mean change, typical error |
| Reis et al. (2019) [58] | Healthy adults  Convenience sample from four fitness centres | n= 56  28 (5) yrs  0% | Resistance exercises  (half squat, 45˚ inclined leg press, leg extension, horizontal bench press, 45˚ inclined bench press, lateral pull down, triceps extension and biceps curl) | Two exercises were randomly assigned to each group and subjects performed four bouts of 4-min constant-intensity at each assigned exercise: 12%, 16%, 20% and 24% 1-RM | Polar V800 (not declared) | none declared | Indirect calorimetry | Bland-Altman plot, correlation, regression |
| Sasaki et al. (2015) [41] | Healthy adults  Convenience sample from University community | n= 20  24 (5) yrs  50% | Three activity clusters;  1. office work, driving, carrying a box, carrying groceries, stairs.  2. cycling, golf, tennis and basketball.  3 dusting, laundry, vacuuming, raking, gardening | 4 mins/activity, driving was 11 mins.  Participants were assigned to one of three routines. | Fitbit Classic (waist) | Sex, age, height and weight | Indirect calorimetry | Bland-Altman plot, correlation, t-test, mean percent difference |
| Woodman et al. (2017) [59] | Healthy adults  Convenience sample from University and surrounding community | n= 28  26 (4) yrs  29% | Sedentary activities  (supine rest, seated rest, computer usage in seated position, folding clothes in seated position)  Activities of daily living  (sweeping floor, treadmill walking climbing stairs, overground walking, overground running, overground cycling outside on a standard bicycle, ergometer cycling) | Supine rest: 10 mins, all other: 5 mins/activity | Basis Peak (wrist)  Garmin VivoFit (wrist)  Withings Pulse (3 devices, wrist, shirt collar, and right hip) | Basis Peak and Garmin VivoFit: Sex, age, height and weight.  Withings Pulse: no information required | Indirect calorimetry | Bland-Altman plot, MAPE, ANOVA, |
| Yavelberg et al. (2018) [49] | Healthy adults  Individuals with Type 1 diabetes  Not declared | n= 5 – 7  25 (8) yrs  Not declared | Resistance exercises  (marching on the spot with high knees, squat with a front sweep using a medicine ball swinging between the legs and over the head, jumping jacks, a quadruped motion (palms flat on the floor, extending 1 arm and opposite leg simultaneously), push-ups, forearm plank, squats with medicine ball placed at chest height shelf) | Exercises ranging from 20 to 60 seconds | Garmin Vivofit2 (wrist)  Mio Fuse (wrist) | None declared | Indirect calorimetry | T-test |

*Supplemental Table 4: Studies using Free-Living Protocols. ICC; intraclass correlation coefficient, MAPE; mean absolute percent error, RMSE; root mean square error, CV; coefficient of variation.*

| **Author (year)** | **Target population & Recruitment** | **N**  **age (SD) or range**  **females/males** | **Types of activities** | **Index device (placement)** | **Specified inputs to index device** | **Criterion** | **Statistical comparison** |
| --- | --- | --- | --- | --- | --- | --- | --- |
| Kinnunen et al. (2019) [60] | Healthy adults    ‘Preferential’ random sample from volunteers participating in another research study | n= 15  30 (6) yrs  0% | 2x14 days free-living. During control period and last 14 days of strength-training condition in experimental study | Polar Active | Body height. Physical Activity Level reported by device multiplied by estimated REE using same equations as for the double labeled water. | Doubly-labelled water | Bland-Altman plot, correlation |
| Murakami et al. (2016) [54] | Healthy adults  Not declared | n= 21  32 (10) yrs  57% | 15 days free-living | Fitbit Flex (wrist)  Jawbone UP24 (wrist)  Misfit Shine (wrist)  Epson Pulsence PS-100 (wrist)  Garmin Vivofit (wrist)  TANITA AM-160 (pocket)  OMRON CaloriScan HJA-403C (pocket)  Withings Pulse O2 (waist) | none declared | Doubly-labelled water | Correlation, mean difference |
| Murakami et al. (2019) [55] | Healthy adults  Not declared | n= 21  32 (10) yrs  57% | 15 days free-living | Fitbit Flex (wrist)  Jawbone UP24 (wrist)  Misfit Shine (wrist)  Epson Pulsence PS-100 (wrist)  Garmin Vivofit (wrist)  TANITA AM-160 (pocket)  OMRON CaloriScan HJA-403C (pocket)  Withings Pulse O2 (waist) | none declared | Doubly-labelled water | MAPE, correlation, mean difference |
| Ramirez-Marrero et al. (2005) [61] | Children  Not declared | n= 12  8 (1) yrs  58% | 7 days free-living | Yamax SW-200 Digiwalker | The step counter was reset every morning after recording the number of steps the preceding day. | Doubly-labelled water | Linear regression, correlation |
| Siddal (2019) [62] | Healthy adults  Convenience sample of British Army officer cadets | n= 20  23 (2) yrs  50% | 10-days of military training.  Training period encompassed a selection of typical military activities, including classroom‐based lessons and military‐specific exercise. | Fitbit Surge HR (wrist) | Sex, age, height and weight | Doubly-labelled water | Bland-Altman plot, equivalence testing, correlation, t-test |

## Supplemental File References

[1] Adam Noah J, Spierer D, Gu J, et al. Comparison of steps and energy expenditure assessment in adults of Fitbit Tracker and Ultra to the Actical and indirect calorimetry. *J Med Eng Technol* 2013; 37: 456–462.

[2] Alsubheen S, George A, Baker A, et al. Accuracy of the vivofit activity tracker. *J Med Eng Technol* 2016; 40: 298–306.

[3] Bai Y, Welk G, Nam Y, et al. Comparison of Consumer and Research Monitors under Semistructured Settings. *Med Sci Sport Exerc* 2016; 48: 151–158.

[4] Bai Y, Hibbing P, Mantis C, et al. Comparative evaluation of heart rate-based monitors: Apple Watch vs Fitbit Charge HR. *J Sport Sci* 2018; 36: 1734–1741.

[5] Bassett DR, Ainsworth BE, Swartz AM, et al. Validity of four motion sensors in measuring moderate intensity physical activity. *Med Sci Sports Exerc* 2000; 32: S471–S480.

[6] Boudreaux B, Hebert E, Hollander D, et al. Validity of Wearable Activity Monitors during Cycling and Resistance Exercise. *Med Sci Sport Exerc* 2018; 50: 624–633.

[7] Chowdhury E, Western M, Nightingale T, et al. Assessment of laboratory and daily energy expenditure estimates from consumer multi-sensor physical activity monitors. *PLoS One* 2017; 12: e0171720.

[8] Compagnat M, Mandigout S, CS B, et al. Validity of wearable actimeter computation of total energy expenditure during walking in post-stroke individuals. *Ann Phys Rehabil Med* 2020; 63: 209–215.

[9] Dannecker K, Sazonova N, Melanson E, et al. A comparison of energy expenditure estimation of several physical activity monitors. *Med Sci Sport Exerc* 2013; 45: 2105–2112.

[10] Diaz KM, Krupka DJ, Chang MJ, et al. Fitbit®: An accurate and reliable device for wireless physical activity tracking. *Int J Cardiol* 2015; 185: 138–140.

[11] Diaz K, Krupka D, Chang M, et al. Validation of the Fitbit One® for physical activity measurement at an upper torso attachment site. *BMC Res Notes* 2016; 9: 213.

[12] Dooley E, Golaszewski N, Bartholomew J. Estimating Accuracy at Exercise Intensities: A Comparative Study of Self-Monitoring Heart Rate and Physical Activity Wearable Devices. *JMIR Mhealth Uhealth* 2017; 5: e34.

[13] Düking P, Giessing L, Frenkel MO, et al. Wrist-worn wearables for monitoring heart rate and energy expenditure while sitting or performing light-to-vigorous physical activity: validation study. *JMIR mHealth uHealth* 2020; 8: e16716.

[14] Falter M, Budts W, Goetschalckx K, et al. Accuracy of Apple Watch Measurements for Heart Rate and Energy Expenditure in Patients With Cardiovascular Disease: Cross-Sectional Study. *JMIR Mhealth Uhealth* 2019; 7: e11889.

[15] Gilgen-Ammann R, Schweizer T, Wyss T. Accuracy of the Multisensory Wristwatch Polar Vantage’s Estimation of Energy Expenditure in Various Activities: Instrument Validation Study. *JMIR Mhealth Uhealth* 2019; 7: e14534.

[16] Godino JG, Wing D, de Zambotti M, et al. Performance of a commercial multi-sensor wearable (Fitbit Charge HR) in measuring physical activity and sleep in healthy children. *PLoS One*; 15. Epub ahead of print 2020. DOI: 10.1371/journal.pone.0237719.

[17] Hendrikx J, Ruijs LS, Cox LGE, et al. Clinical Evaluation of the Measurement Performance of the Philips Health Watch: A Within-Person Comparative Study. *JMIR mHealth uHealth*; 5. Epub ahead of print 2017. DOI: 10.2196/mhealth.6893.

[18] Herkert C, JJ K, EMA van L, et al. Usefulness of Modern Activity Trackers for Monitoring Exercise Behavior in Chronic Cardiac Patients: Validation Study. *JMIR Mhealth Uhealth* 2019; 7: e15045.

[19] Hopkins L, Stacey B, DBT R, et al. Consumer-grade biosensor validation for examining stress in healthcare professionals. *Physiol Rep* 2020; 8: e14454.

[20] Imboden M, Nelson M, Kaminsky L, et al. Comparison of four Fitbit and Jawbone activity monitors with a research-grade ActiGraph accelerometer for estimating physical activity and energy expenditure. *Br J Sport Med* 2018; 52: 844–850.

[21] Johnson M, Turek J, Dornfeld C, et al. Validity of the Samsung Phone S Health application for assessing steps and energy expenditure during walking and running: Does phone placement matter? *Digit Heal* 2016; 2: 2055207616652747.

[22] Kane NA, Simmons MC, John D, et al. Validity of the Nike plus Device During Walking and Running. *Int J Sport Med* 2010; 31: 101–105.

[23] Kendall B, Bellovary B, Gothe NP. Validity of wearable activity monitors for tracking steps and estimating energy expenditure during a graded maximal treadmill test. *J Sports Sci* 2019; 37: 42–49.

[24] LaMunion S, Blythe A, Hibbing P, et al. Use of consumer monitors for estimating energy expenditure in youth. *Appl Physiol Nutr Metab* 2020; 45: 161–168.

[25] Lee J, Kim Y, Welk G. Validity of consumer-based physical activity monitors. *Med Sci Sport Exerc* 2014; 46: 1840–1848.

[26] Leenders N, Nelson TE, Sherman WM. Ability of different physical activity monitors to detect movement during treadmill walking. *Int J Sport Med* 2003; 24: 43–50.

[27] Lemmens P, Sartor F, Cox L, et al. Evaluation of an activity monitor for use in pregnancy to help reduce excessive gestational weight gain. *BMC Pregnancy Childbirth* 2018; 18: 312.

[28] Machač S, Procházka M, Radvanský J, et al. Validation of physical activity monitors in individuals with diabetes: energy expenditure estimation by the multisensor SenseWear Armband Pro3 and the step counter Omron HJ-720 against indirect calorimetry during walking. *Diabetes Technol Ther* 2013; 15: 413–418.

[29] Maddison R, Gemming L, Monedero J, et al. Quantifying Human Movement Using the Movn Smartphone App: Validation and Field Study. *JMIR mHealth uHealth*; 5. Epub ahead of print 2017. DOI: 10.2196/mhealth.7167.

[30] Montoye AHK, Mitrzyk JR, Molesky MJ. Comparative Accuracy of a Wrist-Worn Activity Tracker and a Smart Shirt for Physical Activity Assessment. *Meas Phys Educ Exerc Sci* 2017; 21: 201–211.

[31] Moreno D, Glasheen E, Domingo A, et al. Validity of Caloric Expenditure Measured from a Wheelchair User Smartwatch. *Int J Sport Med* 2020; 41: 505–511.

[32] Nolan M, Mitchell J, Doyle-Baker P. Validity of the Apple iPhone® /iPod Touch® as an accelerometer-based physical activity monitor: a proof-of-concept study. *J Phys Act Heal* 2014; 11: 759–769.

[33] Nuss KJ, Thomson EA, Courtney JB, et al. Assessment of Accuracy of Overall Energy Expenditure Measurements for the Fitbit Charge HR 2 and Apple Watch. *Am J Health Behav* 2019; 43: 498–505.

[34] O’Driscoll R, Turicchi J, Hopkins M, et al. The validity of two widely used commercial and research-grade activity monitors, during resting, household and activity behaviours. *Health Technol (Berl)* 2020; 10: 637–648.

[35] O’Driscoll R, Turicchi J, Hopkins M, et al. Improving energy expenditure estimates from wearable devices: A machine learning approach. *J Sport Sci* 2020; 38: 1496–1505.

[36] Parak J, Uuskoski M, Machek J, et al. Estimating Heart Rate, Energy Expenditure, and Physical Performance With a Wrist Photoplethysmographic Device During Running. *JMIR mHealth uHealth*; 5. Epub ahead of print 2017. DOI: 10.2196/mhealth.7437.

[37] Passler S, Bohrer J, Blöchinger L, et al. Validity of Wrist-Worn Activity Trackers for Estimating VO(2max) and Energy Expenditure. *Int J Env Res Public Heal*; 16. Epub ahead of print 2019. DOI: 10.3390/ijerph16173037.

[38] Price K, Bird S, Lythgo N, et al. Validation of the Fitbit One, Garmin Vivofit and Jawbone UP activity tracker in estimation of energy expenditure during treadmill walking and running. *J Med Eng Technol* 2017; 41: 208–215.

[39] Reddy R, Pooni R, Zaharieva D, et al. Accuracy of Wrist-Worn Activity Monitors During Common Daily Physical Activities and Types of Structured Exercise: Evaluation Study. *JMIR Mhealth Uhealth* 2018; 6: e10338.

[40] Roos L, Taube W, Beeler N, et al. Validity of sports watches when estimating energy expenditure during running. *BMC Sport Sci Med Rehabil* 2017; 9: 22.

[41] Sasaki J, Hickey A, Mavilia M, et al. Validation of the Fitbit wireless activity tracker for prediction of energy expenditure. *J Phys Act Heal* 2015; 12: 149–154.

[42] Shcherbina A, Mattsson C, Waggott D, et al. Accuracy in Wrist-Worn, Sensor-Based Measurements of Heart Rate and Energy Expenditure in a Diverse Cohort. *J Pers Med*; 7. Epub ahead of print 2017. DOI: 10.3390/jpm7020003.

[43] Slootmaker S, Chin A Paw M, Schuit A, et al. Concurrent validity of the PAM accelerometer relative to the MTI Actigraph using oxygen consumption as a reference. *Scand J Med Sci Sport* 2009; 19: 36–43.

[44] Swartz A, Strath S, Miller N, et al. Validity of Physical Activity Monitors in Assessing Energy Expenditure in Normal, Overweight, and Obese Adults. *Open Sport Sci J* 2009; 2: 58–64.

[45] Thiebaud R, Funk M, Patton J, et al. Validity of wrist-worn consumer products to measure heart rate and energy expenditure. *Digit Heal* 2018; 4: 2055207618770322.

[46] Wahl Y, Düking P, Droszez A, et al. Criterion-Validity of Commercially Available Physical Activity Tracker to Estimate Step Count, Covered Distance and Energy Expenditure during Sports Conditions. *Front Physiol* 2017; 8: 725.

[47] Wallen MP, Gomersall SR, Keating SE, et al. Accuracy of Heart Rate Watches: Implications for Weight Management. *PLoS One*; 11. Epub ahead of print 2016. DOI: 10.1371/journal.pone.0154420.

[48] Xie J, Wen D, Liang L, et al. Evaluating the Validity of Current Mainstream Wearable Devices in Fitness Tracking Under Various Physical Activities: Comparative Study. *JMIR Mhealth Uhealth* 2018; 6: e94.

[49] Yavelberg L, Zaharieva D, Cinar A, et al. A Pilot Study Validating Select Research-Grade and Consumer-Based Wearables Throughout a Range of Dynamic Exercise Intensities in Persons With and Without Type 1 Diabetes: A Novel Approach. *J Diabetes Sci Technol* 2018; 12: 569–576.

[50] Zhang P, Burns RD, Fu Y, et al. Agreement between the Apple Series 1, LifeTrak Core C200, and Fitbit Charge HR with Indirect Calorimetry for Assessing Treadmill Energy Expenditure. *Int J Environ Res Public Health*; 16. Epub ahead of print 2019. DOI: 10.3390/ijerph16203812.

[51] Zhang P, Godin S, Owens M. Measuring the validity and reliability of the Apple Watch as a physical activity monitor. *J Sport Med Phys Fit* 2019; 59: 784–790.

[52] Brugniaux J, Niva A, Pulkkinen I, et al. Polar Activity Watch 200: a new device to accurately assess energy expenditure. *Br J Sport Med* 2010; 44: 245–249.

[53] Morris C, Wessel P, Tinius R, et al. Validity of Activity Trackers in Estimating Energy Expenditure During High-Intensity Functional Training. *Res Q Exerc Sport* 2019; 90: 377–384.

[54] Murakami H, Kawakami R, Nakae S, et al. Accuracy of Wearable Devices for Estimating Total Energy Expenditure: Comparison With Metabolic Chamber and Doubly Labeled Water Method. *JAMA Intern Med* 2016; 176: 702–703.

[55] Murakami H, Kawakami R, Nakae S, et al. Accuracy of 12 wearable devices for estimating physical activity energy expenditure using a metabolic chamber and the doubly labeled water method: validation study. *JMIR mHealth uHealth* 2019; 7: e13938.

[56] Nelson M, Kaminsky L, Dickin D, et al. Validity of Consumer-Based Physical Activity Monitors for Specific Activity Types. *Med Sci Sport Exerc* 2016; 48: 1619–1628.

[57] Reeve M, Pumpa K, Ball N. Accuracy of the SenseWear Armband Mini and the BodyMedia FIT in resistance training. *J Sci Med Sport* 2014; 17: 630–634.

[58] Reis VM, Vianna JM, Barbosa TM, et al. Are wearable heart rate measurements accurate to estimate aerobic energy cost during low-intensity resistance exercise? *PLoS One*; 14. Epub ahead of print 2019. DOI: 10.1371/journal.pone.0221284.

[59] Woodman JA, Crouter SE, Bassett DR, et al. Accuracy of Consumer Monitors for Estimating Energy Expenditure and Activity Type. *Med Sci Sports Exerc* 2017; 49: 371–377.

[60] Kinnunen H, Häkkinen K, Schumann M, et al. Training-induced changes in daily energy expenditure: Methodological evaluation using wrist-worn accelerometer, heart rate monitor, and doubly labeled water technique. *PLoS One* 2019; 14: e0219563.

[61] Ramirez-Marrero FA, Smith BA, Sherman WM, et al. Comparison of methods to estimate physical activity and energy expenditure in African American children. *Int J Sport Med* 2005; 26: 363–371.

[62] Siddall A, Powell S, Needham-Beck S, et al. Validity of energy expenditure estimation methods during 10 days of military training. *Scand J Med Sci Sport* 2019; 29: 1313–1321.
